# Condensation of acrylonitrile and aryl acetonitrile: construction of α-amino-β-cyano cyclohexene skeletons†

**DOI:** 10.1039/d2ra04936h

**Published:** 2022-10-18

**Authors:** Wei Zhang, Chuan-Su Tang, Shi-Qun Xiang

**Affiliations:** Key Laboratory of Preparation and Application of Environmental Friendly Materials, Jilin Normal University, Ministry of Education Changchun 130103 China xiangshq@jlnu.edu.cn; College of Chemistry, Jilin Normal University Siping 136000 China; Chongqing University of Science and Technology Chongqing 401331 China; Jiangjin No. 5 Middle School Chongqing 402260 China

## Abstract

A representative condensation of acrylonitrile and aryl acetonitrile has been reported for the synthesis of α-amino-β-cyano cyclohexene. The reaction was carried out mildly in an open environment at room temperature. The scope and versatility of the method have been demonstrated with 20 examples, containing highly active ethynyl groups. Further applications for 4-aminopyrimidine compounds were performed. A mechanism was proposed, involving Michael additions between acrylonitrile and aryl acetonitriles as well as intramolecular condensation.

## Abstract 2

A condensation reaction between acrylonitrile and benzyl cyanide for the synthesis of α-amino-β-cyano cyclohexene was reported. The reaction could be carried out mildly with high atomic efficiency to build the cyclohexene skeleton.

## Graphical abstract

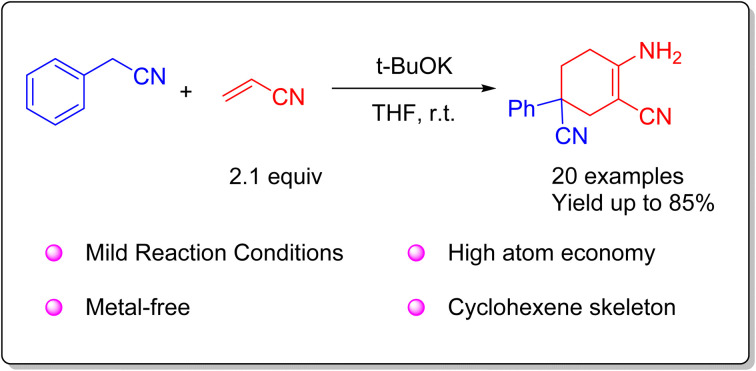

Multi-substituted cyclohexenes are important building blocks found in natural products, anti-influenza drugs and spices.^1^ Oseltamivir, vitamin A, dynascone and beta-ionone are widely used representative molecules.^2^ The well-known anti-flu drug oseltamivir also possesses a cyclohexene skeleton (Fig. 1a). Unlike the *N*-containing heterocyclic skeletons of pyridines, indoles and aminopyrimidines, little attention has been paid to the construction of cyclohexene skeletons. Shikimic acid, the raw compound for oseltamivir, is generally obtained from phytoextraction or biological fermentation.^3^ Further functionalization or modification is required considering the limited candidates restricted by these approaches.

**Fig. 1:**
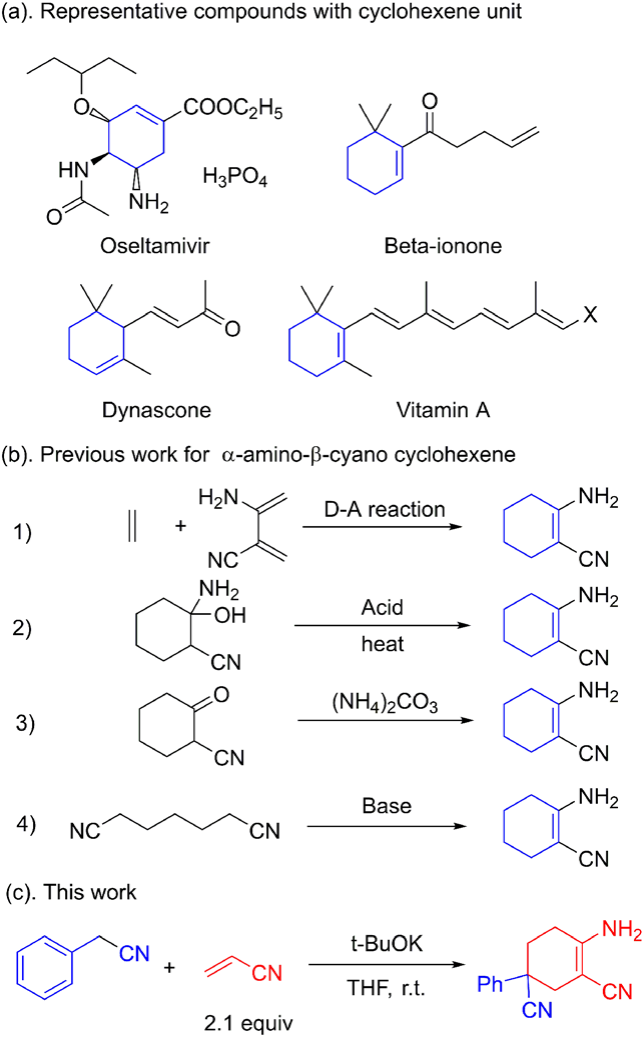
Selected examples and previous works.

The Diels–Alder reaction is commonly applied in the construction of the cyclohexene skeleton as a standard template.^4^ However, to the best of our knowledge, this [4 + 2] ring addition for the α-amino-β-cyano cyclohexene skeleton is unpractical. The acid-catalyzed dehydration of cyclohexanol is also used for the synthesis of cyclohexene.^5^ The use of acids and rigorous reaction conditions leads to tedious post-processing steps. The condensation between ammonium and α-cyano cyclohexanone is thought to be feasible in the construction of the cyclohexene skeleton. However, the use of expensive α-cyano cyclohexanone makes this strategy impracticable.^6^ The synthesis of α-amino-β-cyano cyclohexene *via* Thorpe–Ziegler condensation is commonly regarded as an intramolecular exception in the condensation of pimelic dinitrile (Fig. 1b).^7^ This reaction for building a cyclohexene skeleton is restricted by the fact that there are only a few active sites for further extension. Unlike the common synthetic approach using β-enaminonitrile, α-amino-β-cyano cyclohexene has a *Z*-configuration, which has high energy and high reactivity. The multifunctional unit has proved to be a key construction block in the synthesis of heterocyclic compounds such as pyrimidine, pyridine, pyrrole, pyrazole and imidazole.^8^

Herein, we present a novel synthetic cyclic strategy towards 2,4-dicyano-1-amino cyclohexene skeletons. This strategy contains Michael additions between acrylonitrile and aryl acetonitriles as well as intramolecular condensation.^9^ We carried out the reaction mildly in an open environment at room temperature. Moreover, further applications of 4-aminopyrimidine compounds were performed.

We began our initial study using aryl acetonitrile (1a) and acrylonitrile (2) as benchmark substrates for condensation. All the reactions were carried out at room temperature in air. We optimized the reaction conditions by changing the ingredients or conditions such as base, solvent and time to find the most suitable conditions (Table 1). Using the substrates 1a (0.4 mmol) and 2 (0.84 mmol), potassium *tert*-butoxide (1.2 mmol) as a base and THF as a solvent, the reaction gave the best yield after stirring for 2 h (entry 1). Bases were scanned and potassium *tert*-butoxide was found to be the best option. Considering the poor performance of potassium carbonate (K_2_CO_3_) and potassium hydroxide (KOH), the solubility of the base in organic solvent would have a great effect on the reaction (entries 2–3). Reactions with soluble bases such as 1,8-diazabicyclo[5.4.0]-undec-7-ene (DBU) and lithium hexamethyldisilazide (LiHMDS) afforded more products (entries 4–5). LiHMDS was thought to be excessively alkaline in the deprotonation step and could therefore not participate in the final cyclization process. We then scanned the effect of solvents. Among the tested solvents, esters such as THF and 1,4-dioxane performed better than other commonly used solvents (entries 1, 8–11). However, the reaction seemed to be intolerable in aqueous conditions (entry 12).

**Table 1:** Optimization of the formation of 4-phenyl-2,4-dicyano-1-aminocyclohexene^a^

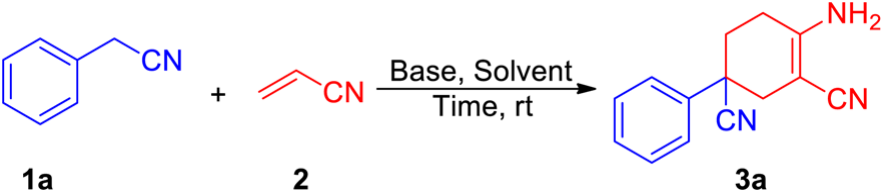				
En.	Base	Solvent	Time	Yield^b^
1	^***t***^ **BuOK**	THF	2 h	84
2	**K**_**2**_ **CO**_**3**_	THF	2 h	0
3	**KOH**	THF	2 h	Trace
4	**DBU**	THF	2 h	42
5	**LiHMDS**	THF	2 h	17
6^c^	^*t*^BuOK	THF	2 h	60
7^d^	^*t*^BuOK	THF	2 h	74
8	^*t*^BuOK	**Toluene**	2 h	56
9	^*t*^BuOK	**Dioxane**	2 h	80
10	^*t*^BuOK	**Acetonitrile**	2 h	67
11	^*t*^BuOK	**DMF**	2 h	53
12	^*t*^BuOK	**Water**	2 h	0
13	^*t*^BuOK	THF	**0.25 h**	16
14	^*t*^BuOK	THF	**0.5 h**	43
15	^*t*^BuOK	THF	**4 h**	84

a Reaction conditions: 1a (0.4 mmol), 2 (0.84 mmol), base (1.2 mmol) and solvent (3 mL) at room temperature.

b Isolated yield.

c Base (1 equiv.).

d Base (2 equiv.).

With the best conditions of this reaction in hand, we sought to investigate the generality and functional group compatibility of phenylacetonitrile (1a) under the established conditions, and the results are presented in Table 2. The production efficiency of phenylacetonitrile derivatives was discussed based on the electronic effect, steric effect and synergic effect of the functional groups on phenylacetonitrile. The *para*-position of phenylacetonitrile (1a) was chosen to discuss the electronic effect of different types of functional groups in this reaction. When the substituent group on the *para*-position of the phenyl ring was an electron-donating group (EDG), such as methyl, methoxyl and *tert*-butyl, the products were obtained in similar yield (3a–3e, 82–85%). This showed that the EDG had little effect on the production of compounds. In addition, the results show that the electron-withdrawing group (EWG) at the same position as the EDG is harmful to this reaction and the yield would decrease as the EWG became stronger (3f–3h, 41–77%). Then, the effect of steric hindrance on the reaction was studied by using methoxyl (3c, 3i), chloro (3g, 3j, 3l) and fluoro groups (3h, 3k, 3m) in the *para*-, *meta*- and *ortho*-positions, respectively. The results show that the yields of the reaction decreased dramatically from the substitution of the *para*-position to the *ortho*-position. This conclusion was in accordance with the fact that polysubstituted phenylacetonitriles (such as dimethyl, dimethoxyl and dimethylenedioxyl) provided the corresponding products in good yields (3n–3o, 73–78%). Finally, we investigated other types of acetonitrile compounds such as pyridyl acetonitriles and 1-naphthyl acetonitrile. 2-Pyridyl acetonitrile afforded a different imine product. This was probably due to the hydrogen bond between the 2-pyridyl N atom and the H atom of the cyclohexane skeleton. These results show that these substrates could react and afford the target products with a decent yield. Moreover, the conjugate phenylacetonitrile with a highly reactive acetenyl group was investigated under the same reaction conditions and afforded the product 3t in a yield of 54%. The isolation of 3t proved that substrates with unsaturated functional groups may also be adaptable for the mild reaction system.

**Table 2:** Reaction scope with various phenylacetonitrile derivatives^a^^,^^b^

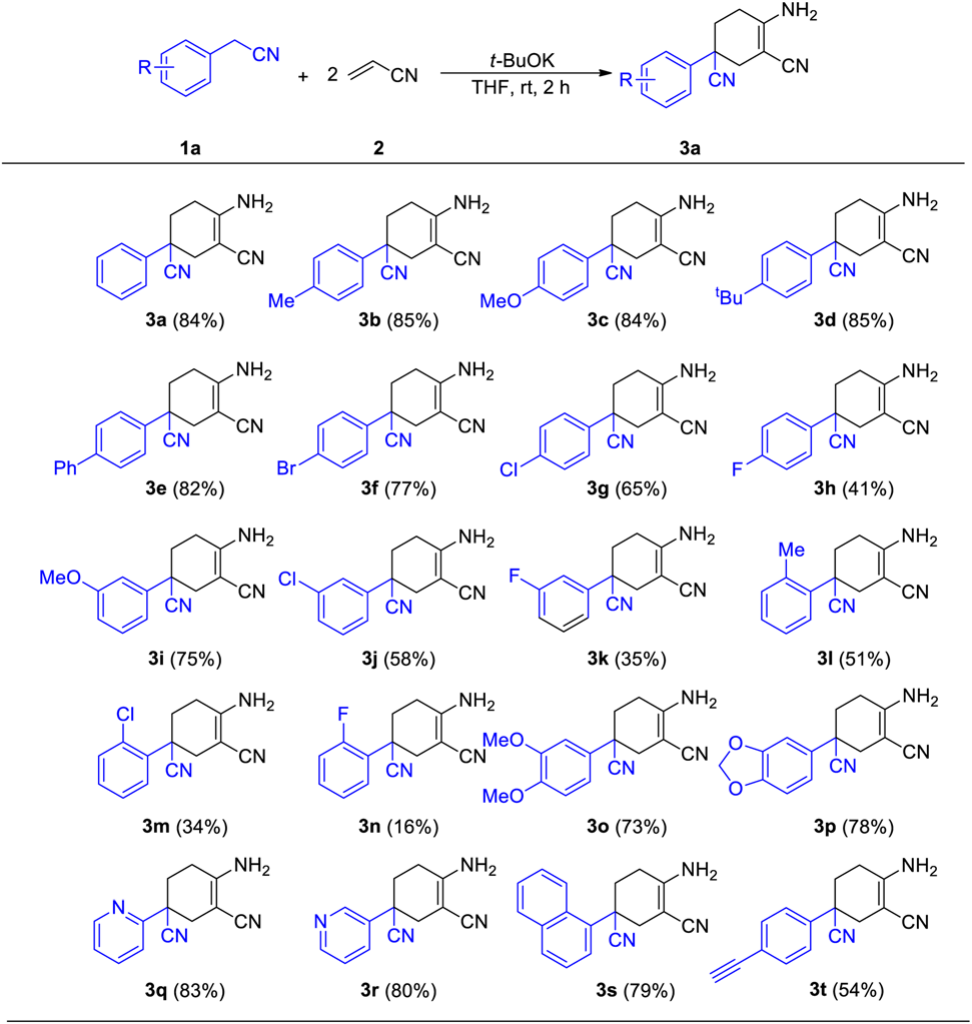

a Reaction conditions: 1a (0.4 mmol), 2 (0.84 mmol), *t*-BuOK (1.2 mmol) and solvent (3 mL) at room temperature for 2 h.

b Isolated yield.

In addition, further studies were performed for a better understanding of the reaction, and a by-product 3n′ was isolated under the standard reaction conditions for the synthesis of 3n. 3-(2-Fluorophenyl)pentane-1,3,5-tricarbonitrile was thought to be one of the intermediates for the reaction (Scheme 3). Further utilization of compound 3n′ could afford the final product 3n in a yield of 53%. Moreover, the synthetic utility of our reaction was examined by performing gram-scale experiments. The reaction of phenylacetonitrile (1a) and acrylonitrile (2) on a 5.0 g gram scale under the standard conditions generated the compound 3a in 71% yield (Scheme 1c). For further extension, we found that the product 3a and benzonitrile (4a) could be converted to the 4-aminopyrimidines compound 5a (Scheme 2a). The same reaction was carried out using three other compounds (4b, 4c and 4d) as the starting materials (Scheme 2b–d). The crystal structures of compounds 3a and 5a were determined by X-ray crystallography (Fig. 2).

**Scheme 1:**
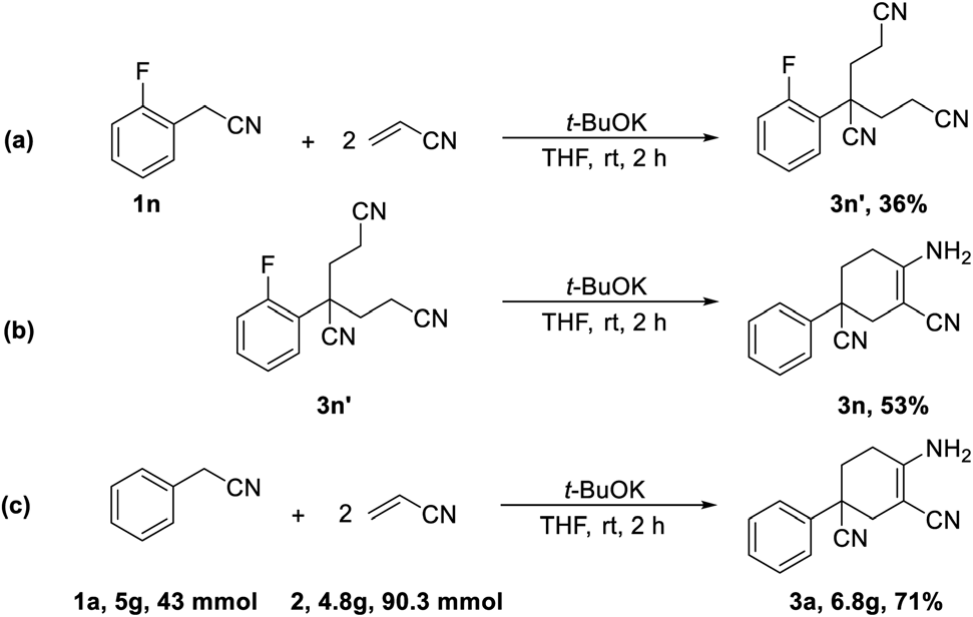
Mechanistic studies and gram-scale experiments.

**Scheme 2:**
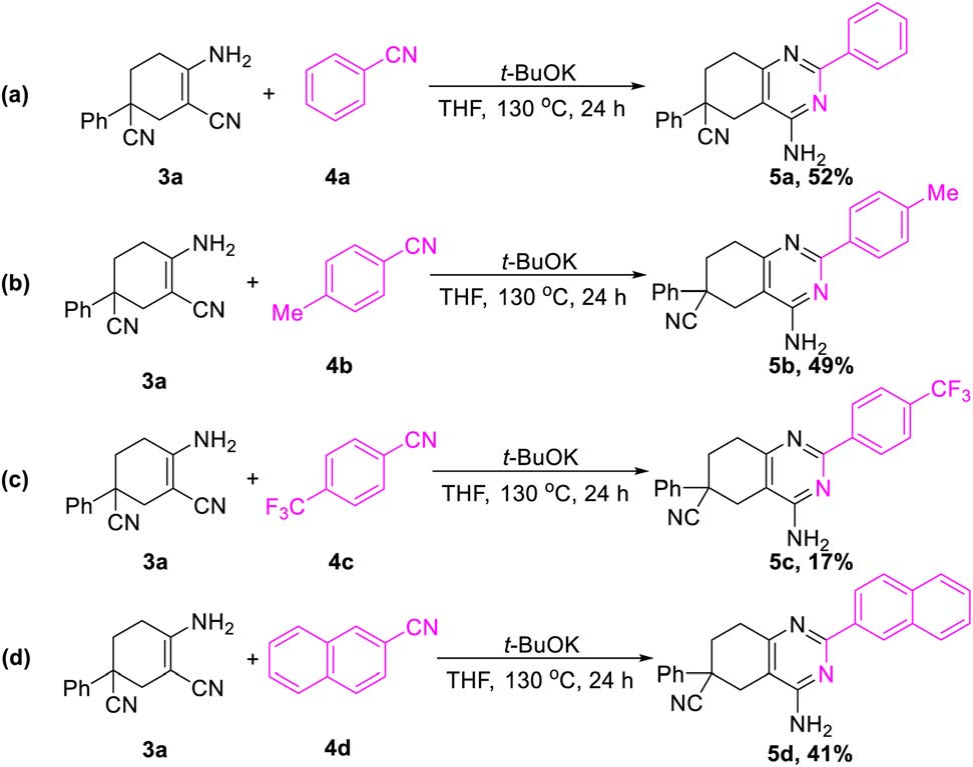
Applications of the products.

**Fig. 2:**
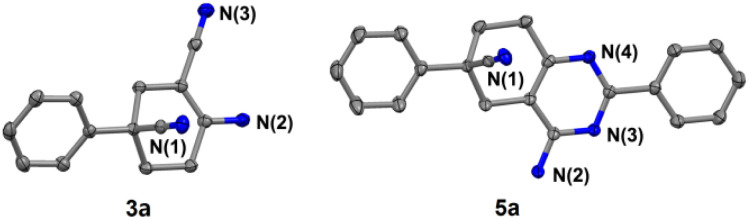
Crystal structures of compounds 3a (CCDC 2158344) and 5a (CCDC 2158132).

A plausible mechanism has been depicted in Scheme 3. In the presence of a base, the methylene group of aryl acetonitrile loses a proton and turns into a cyano-alkylide anion (a). Nucleophilic attack by the R–CH^−^–CN anion results in the formation of 4-phenyl-4-cyano-butyl nitrile (b) with the consumption of a molecule of acrylonitrile. Another molecule of acrylonitrile is consumed for the synthesis of the subsequent intermediate 4-phenyl-4-cyano-pimelic dinitrile (c).^10^ The binitrile intermediate (c) then performs a base-promoted intramolecular condensation (Scheme 1b). With the participation of the base, intermediate (c) undergoes a deprotonation process to afford the anion intermediate (d). The nucleophilic attack from the alpha-carbon anion to the cyano group leads to the formation of imine species and ultimately results in the final product 3a.

**Scheme 3:**
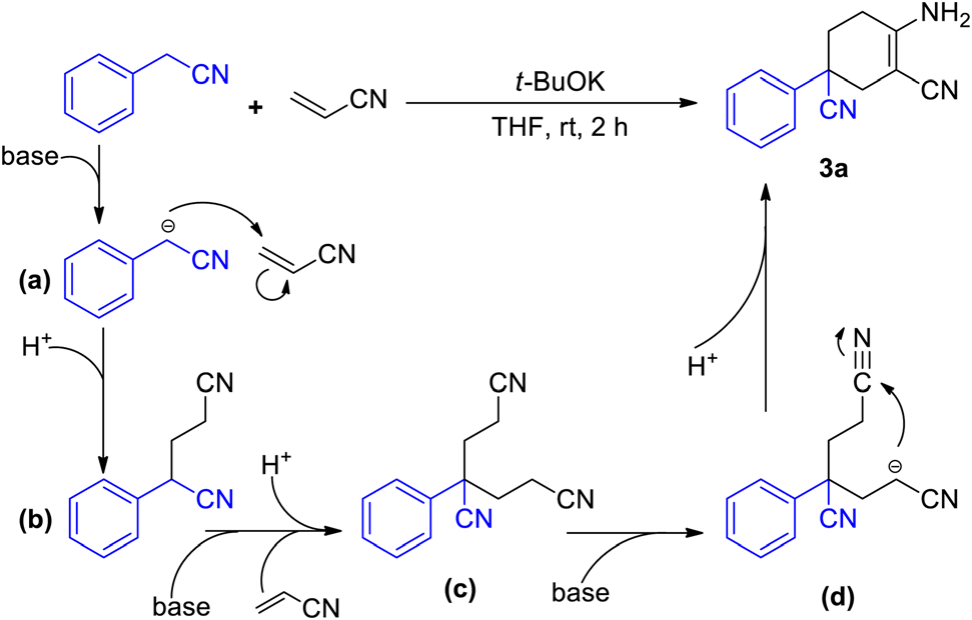
Possible reaction mechanism.

## Conclusions

Herein, we report an example of the condensation of acrylonitrile and aryl acetonitrile for the synthesis of α-amino-β-cyano cyclohexene. The reaction could be carried out mildly at room temperature. Most of the final products could be isolated without column chromatography. Further applications were performed for multi-substituted 4-aminopyrimidines and a plausible base-induced nucleophilic mechanism was proposed.

## Author contributions

W. Z.: conceptualization, data curation, formal analysis, investigation, methodology, visualization, writing-original draft. C. T.: data curation. S. X.: funding acquisition, project administration, resources, software, supervision, validation, writing-review & editing.

## Conflicts of interest

There are no conflicts to declare.

## Supplementary Material

RA-012-D2RA04936H-s001

RA-012-D2RA04936H-s002
