# Supplementary material for: Condensation of acrylonitrile and aryl acetonitrile: construction of α-amino-β-cyano cyclohexene skeletons
Source: RSC Adv. 2022 Oct 18;12(46):29840–3. doi: 10.1039/d2ra04936h (PMC9578340; doi:10.1039/d2ra04936h)
Supplement: RA-012-D2RA04936H-s001 [file RA-012-D2RA04936H-s001.pdf]

Electronic Supplementary Material (ESI) for RSC Advances.
This journal is © The Royal Society of Chemistry 2022

Supporting Information

Condensation of Acrylonitrile and Benzyl Cyanide: Construction of *α*-Amino-*β*-cyano Cyclohexene Skeletons

Wei Zhang,[c] Chuan-Su Tang,[d] Shi-Qun Xiang [a,b,*]

[a]*Key Laboratory of Preparation and Application of Environmental Friendly Materials (Jilin Normal University), Ministry of Education, Changchun, 130103, China.*

[b]*College of Chemistry, Jilin Normal University, Siping, 136000, China.*

[c] *Chongqing university of Science and Technology, Chongqing, 401331, China.*

[d] *Jiangjin No. 5 Middle School, Chongqing, 402260, China.*

**To whom correspondence should be addressed. E-mail: xiangshq@jlnu.edu.cn*

Table of contents:

1. General information ··· S2
2. Experimental procedure ··· S2
3. Characterization data for the products ··· S4
4. X-ray structure determinations ··· S11
5. Crystallographic data of compounds ··· S12
6. ^{1}H NMR and ^{13}C NMR spectra of compounds ··· S13
7. References ··· S38

1. General Information

Chemicals. Unless otherwise stated, commercial grade chemicals were used without further purification. Tetrahydrofuran, 1,2-dimethoxyethane, dioxane and toluene were distilled over sodium under N_2. Volume reduction and drying steps were performed in *vacuo*.

General Physical Measurements. ^{1}H NMR and ^{13}C NMR spectra were recorded on Bruker Avance III and chemical shifts were expressed in δ ppm values with reference to tetramethylsilane (TMS) as internal standard. HR-MS (ESI) spectra were obtained using a Bruker Impact II quardrupole time off light mass spectrometer. The single crystal data were collected on an Oxford Diffraction Supernova dual diffractometer equipped with an Oxford Cryostream 700 low-temperature apparatus.

2. Experimental procedure

2.1 General experimental procedure without column chromatography for th e synthesis of *4-amino-1,2,5,6-tetrahydro-[1,1'-biphenyl]-1,3-dicarbonitrile*s (3a-3g, 3i, 3j, 3l, 3o, 3p, 3q, 3r, 3s, 3t).

A mixture of phenylacetonitrile **1** (0.4 mmol), acrylonitrile (0.84 mmol) and potassium *tert*-butoxide (*t*-BuOK) (1.2 mmol) in tetrahydrofuran (3.0 mL) was stirred at room temperature for 2 h. After cooling down to room temperature, the mixture was added with 1 M HCl solution to adjust the solution to pH < 3 and filtered. The acidic solution was extracted with dichloromethane (5 mL). The aqueous solution was then added with saturated sodium hydroxide aqueous solution to pH = 10. The basic solution was then extracted with chloroform (20 mL X 3). The organic layers were combined and dried with Na_2SO_4. The dried mixture was then concentrated under reduced pressure. The products were collected and dried in vacuo.

2.2 General experimental procedure for the synthesis of *4-amino-1,2,5,6-tetr ahydro-[1,1'-biphenyl]-1,3-dicarbonitrile* (3).

A mixture of phenylacetonitrile **1** (0.4 mmol), acrylonitrile (0.84 mmol) and potassium *tert*-butoxide (*t*-BuOK) (1.2 mmol) in tetrahydrofuran (3.0 mL) was stirred at room temperature for 2 h. The mixture was diluted with dichloromethane (10 mL), filtered through a pad of silica gel, and concentrated under reduced pressure. The crude product was purified on a silica gel column eluted with petroleum ether/ethyl acetate (2:1 v/v) to afford the products **3a-3t**.

2.3 General experimental procedure for the synthesis of *3-(2-fluorophenyl)pe ntane-1,3,5-tricarbonitrile* (3n').

A mixture of phenylacetonitrile **1n** (0.4 mmol), acrylonitrile (0.84 mmol) and

potassium *tert*-butoxide (*t*-BuOK) (1.2 mmol) in tetrahydrofuran (3.0 mL) was stirred at room temperature for 2 h. The mixture was diluted with dichloromethane (10 mL), filtered through a pad of silica gel, and concentrated under reduced pressure. The crude product was purified on a silica gel column eluted with petroleum ether/ethyl acetate (3:1 v/v) to afford the products **3n'** as colorless solid in a yield of 36% (34.7 mg).

2.4 General experimental procedure for the synthesis of *3-(2-fluorophenyl)pentane-1,3,5-tricarbonitrile* (3n).

A mixture of **3n'** (0.12 mmol, 29.0 mg) and potassium *tert*-butoxide (*t*-BuOK) (0.12 mmol, 13.5 mg) in tetrahydrofuran (1.0 mL) was stirred at room temperature for 2 h. The mixture was diluted with dichloromethane (10 mL), filtered through a pad of silica gel, and concentrated under reduced pressure. The crude product was purified on a silica gel column eluted with petroleum ether/ethyl acetate (2:1 v/v) to afford the products **3n** in a yield of 53% (15.3 mg).

2.5 Gram scale experimental procedure for the synthesis of *4-amino-1,2,5,6-tetrahydro-[1,1'-biphenyl]-1,3-dicarbonitrile* (3a).

A mixture of phenylacetonitrile **1a** (43.0 mmol, 5.0 g), acrylonitrile (90.3 mmol, 4.8 g) and potassium *tert*-butoxide (*t*-BuOK) (129.0 mmol, 15.8 g) in tetrahydrofuran (300.0 mL) was stirred at room temperature for 2 h. After cooling down to room temperature, the mixture was added with saturated HCl solution to adjust the solution to pH < 3 and filtered. The acidic solution was concentrated under reduced pressure. The concentrated solution was separated and then exacted with chloroform for several times. The combined organic layers were collected and the solvents were and concentrated under reduced pressure. The product **3a** was collected and dried in vacuo to a yield of 71% (6.8 g).

2.6 General experimental procedure for the synthesis of 4-*amino-5,6,7,8-tetra-hydroquinazoline-6-carbonitrile.*

A mixture of 4-amino-1,2,5,6-tetrahydro-[1,1'-biphenyl]-1,3-dicarbonitrile **3** (0.20 mmol), benzonitrile derivatives **4** (0.20 mmol) and potassium tert-butoxide (*t*-BuOK) (0.25 mmol) in tetrahydrofuran (1.0 mL) was stirred at 130 °C for 24 h in N_2. After cooling to room temperature, the reaction mixture was diluted with dichloromethane (10 mL), filtered through a pad of silica gel, and concentrated under reduced pressure. The crude product was purified on a silica gel column eluted with petroleum ether/ethyl acetate (4:1 to 1:1 v/v) to afford the products **5a-5d**.

3. Characterization Data for the Products

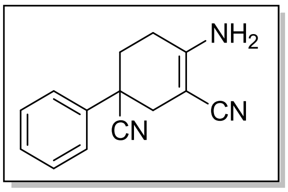

4-amino-5,6-dihydro-[1,1'-biphenyl]-1,3(2H)-dicarbonitrile (3a): Yield, 84% (75.0 mg); colorless solid; melting point, 142-144 °C; ^{1}H NMR (400 MHz, $CDCl_3$) δ 7.49 (d, J = 7.4 Hz, 2H), 7.44 (t, J = 7.2 Hz, 2H), 7.41 – 7.35 (m, 1H), 4.66 (s, 2H), 2.76 (dd, J = 33.9, 15.5 Hz, 3H), 2.39 (d, J = 19.0 Hz, 1H), 2.34 – 2.16 (m, 2H) ^{13}C NMR (101 MHz, $CDCl_3$) δ 155.24 (s), 138.49 (s), 129.23 (s), 128.59 (s), 125.59 (s), 121.70 (s), 119.35 (s), 71.04 (s), 40.93 (s), 35.97 (s), 31.35 (s), 26.33 (s). HR-MS (ESI-TOF) m/z $[M+Na]^+$ calcd. for $C_{14}H_{13}N_3Na$, 246.1007; found, 246.1005. IR (neat) 3430, 3332, 3194, 2187, 2170, 1614, 1453, 1400, 1160 cm^{-1}.

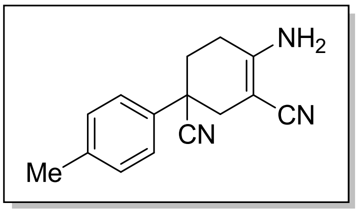

4-amino-4'-methyl-5,6-dihydro-[1,1'-biphenyl]-1,3(2H)-dicarbonitrile (3b): Yield, 85% (80.7 mg); colorless solid; melting point, 140-142 °C; ^{1}H NMR (400 MHz, $CDCl_3$) δ 7.37 (d, J = 8.3 Hz, 2H), 7.24 (d, J = 8.1 Hz, 2H), 4.57 (s, 2H), 2.38 (s, 3H), 2.43 – 2.33 (m, 4H), 2.29 (ddd, J = 11.6, 5.9, 2.9 Hz, 1H), 2.20 (ddd, J = 13.5, 11.2, 5.6 Hz, 1H). ^{13}C NMR (101 MHz, $CDCl_3$) δ 154.97 (s), 138.50 (s), 135.50 (s), 129.86 (s), 125.46 (s), 121.80 (s), 119.27 (s), 71.48 (s), 40.54 (s), 35.95 (s), 31.45 (s), 26.38 (s), 21.03 (s). HR-MS (ESI-TOF) m/z $[M+Na]^+$ calcd. for $C_{15}H_{15}N_3Na$, 260.1163; found, 260.1159. IR (neat) 3456, 3356, 3237, 2927, 2241, 2183, 1241, 1193, 846,813 cm^{-1}.

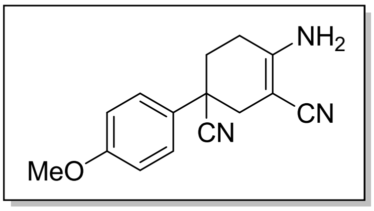

4-amino-4'-methoxy-5,6-dihydro-[1,1'-biphenyl]-1,3(2H)-dicarbonitrile (3c): Yield, 84% (85.1 mg); colorless solid; melting point, 146-148 °C; ^{1}H NMR (400 MHz, $CDCl_3$) δ 7.43 – 7.36 (m, 2H), 6.97 – 6.92 (m, 2H), 4.56 (s, 2H), 3.84 (s, 3H), 2.83 – 2.65 (m, 3H), 2.41 – 2.24 (m, 2H), 2.18 (ddd, J = 13.3, 11.0, 5.6 Hz, 1H). ^{13}C NMR (101 MHz, $CDCl_3$) δ 159.53 (s), 154.94 (s), 130.39 (s), 126.79 (s), 121.88 (s), 119.25 (s), 114.47 (s), 71.48 (s), 55.42 (s), 40.12 (s), 36.01 (s), 31.56 (s), 26.37 (s). HR-MS (ESI-TOF) m/z $[M+Na]^+$ calcd. for $C_{15}H_{15}N_3ONa$, 276.1112; found, 276.1108. IR (neat) 3453, 3359, 2931, 2854, 2242, 2183, 1643, 1413, 1033, 828 cm^{-1}.

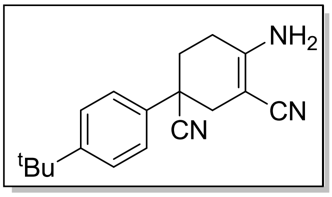

4-amino-4'-(tert-butyl)-5,6-dihydro-[1,1'-biphenyl]-1,3(2H)-dicarbonitrile (3d): Yield, 85% (95.0 mg); colorless solid; melting point, 138-140 °C; ^{1}H NMR (400 MHz, $CDCl_3$) δ 7.48 – 7.38 (m, 4H), 4.50 (s, 2H), 2.84 – 2.68 (m, 3H), 2.42 – 2.21 (m, 3H), 1.35 (s, 9H). ^{13}C NMR (101 MHz, $CDCl_3$) δ 154.71 (s), 151.68 (s), 135.40 (s), 126.14 (s), 125.28 (s), 121.71 (s), 119.13 (s), 71.87 (s), 40.49 (s), 35.94 (s), 31.43 (s), 31.24 (s), 29.73 (s), 26.41 (s). HR-MS (ESI-TOF) m/z $[M+Na]^+$ calcd. for $C_{18}H_{21}N_3Na$, 302.1633; found, 302.1618. IR (neat) 3346, 2958, 2925, 2854, 2187, 1563, 1412, 1020, 928, 830 cm^{-1}.

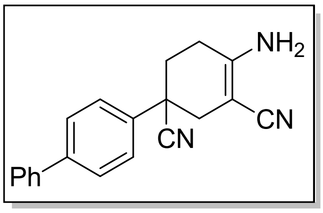

4-amino-5,6-dihydro-[1,1':4',1''-terphenyl]-1,3(2H)-dicarbonitrile (3e): Yield, 82% (98.2 mg); colorless solid; melting point, 201-203 °C;^{1}H NMR (400 MHz, $CDCl_3$) δ 7.69 – 7.64 (m, 2H), 7.64 – 7.59 (m, 2H), 7.59 – 7.54 (m, 2H), 7.52 – 7.46 (m, 2H), 7.41 (ddd, J = 7.3, 3.8, 1.2 Hz, 1H), 4.57 (s, 2H), 2.90 – 2.72 (m, 3H), 2.46 – 2.24 (m, 3H). ^{13}C NMR (101 MHz, $CDCl_3$) δ 154.85 (s), 141.55 (s), 139.92 (s), 137.34 (s), 128.93 (s), 127.95 – 127.87 (m), 127.83 (d, J = 24.4 Hz), 127.09 (s), 126.06 (s), 121.62 (s), 119.17 (s), 71.59 (s), 40.70 (s), 35.94 (s), 31.48 (s), 26.39 (s). HR-MS (ESI-TOF) m/z $[M+Na]^+$ calcd. for $C_{20}H_{17}N_3Na$, 322.1320; found, 322.1317. IR (neat) 3435, 3348, 3244, 2924, 2234, 2187, 1655, 1410, 833, 767 cm^{-1}.

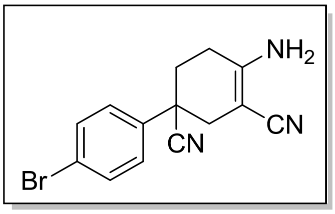

4-amino-4'-bromo-5,6-dihydro-[1,1'-biphenyl]-1,3(2H)-dicarbonitrile (3f): Yield, 77% (93.0 mg); colorless solid; melting point, 144-146 °C; ^{1}H NMR (400 MHz, $CDCl_3$) δ 7.58 (d, J = 8.7 Hz, 2H), 7.38 (t, J = 7.9 Hz, 2H), 4.55 (s, 2H), 2.75 (ddd, J = 22.4, 21.3, 11.1 Hz, 4H), 2.30 (ddd, J = 30.1, 16.6, 3.8 Hz, 3H). ^{13}C NMR (101 MHz, $CDCl_3$) δ 154.67 (s), 137.49 (s), 135.02 (s), 132.39 (s), 127.37 (s), 122.76 (s), 121.12 (s), 71.42 (s), 35.81 (s), 31.39 (s), 26.27 (s). HR-MS (ESI-TOF) m/z $[M+Na]^+$ calcd. for $C_{14}H_{12}BrN_3Na$, 324.0112; found, 324.0109. IR (neat) 3453, 3358, 2853, 2926, 2243, 2182, 1641, 1412, 1162, 846 cm^{-1}.

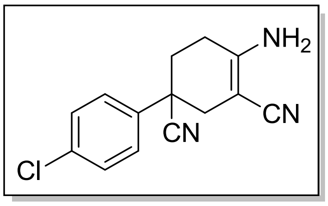

4-amino-4'-chloro-5,6-dihydro-[1,1'-biphenyl]-1,3(2H)-dicarbonitrile (3g): Yield, 65% (67.0 mg); colorless solid; melting point, 155-159 °C;^{1}H NMR (400 MHz, $CDCl_3$) δ 7.46 – 7.39 (m, 4H), 4.56 (s, 2H), 2.84 – 2.65 (m, 3H), 2.42 – 2.13 (m, 3H). ^{13}C NMR (101 MHz, $CDCl_3$) δ 154.72 (s), 136.95 (s), 134.66 (s), 129.43 (s), 127.07 (s), 121.21 (s), 118.97 (s), 71.37 (s), 40.53 (s), 35.88 (s), 31.43 (s), 26.28 (s). HR-MS (ESI-TOF) m/z $[M+Na]^+$ calcd. for $C_{14}H_{12}ClN_3Na$, 280.0617; found, 280.0613. IR (neat) 3453, 3358, 3233, 2925, 2244, 2182, 1642, 1413, 1097, 825 cm^{-1}.

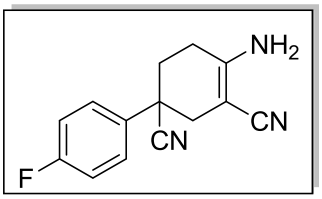

4-amino-4'-fluoro-5,6-dihydro-[1,1'-biphenyl]-1,3(2H)-dicarbonitrile (3h): Yield, 41% (39.5 mg); colorless solid; melting point, 119-121 °C;^{1}H NMR (400 MHz, $CDCl_3$) δ 7.50 – 7.44 (m, 2H), 7.17 – 7.10 (m, 2H), 4.53 (s, 2H), 2.86 – 2.66 (m, 3H), 2.42 – 2.27 (m, 2H), 2.25 – 2.17 (m, 1H). ^{13}C NMR (101 MHz, $CDCl_3$) δ 154.65 (s), 153.23 (d, J = 237.0 Hz), 134.26 (s), 127.46 (d, J = 8.0 Hz), 121.41 (s), 118.96 (s), 116.23 (d, J = 21.0 Hz), 71.59 (s), 40.36 (s), 36.07 (s), 31.58 (s), 26.34 (s). HR-MS (ESI-TOF) m/z $[M+Na]^+$ calcd. for $C_{14}H_{12}FN_3Na$, 264.0912; found, 264.0909. IR (neat) 3439, 3925, 2852, 2240, 2184, 1640, 1563, 1413, 1099, 831 cm^{-1}.

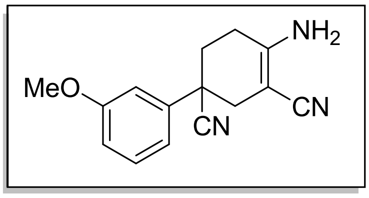

4-amino-3'-methoxy-5,6-dihydro-[1,1'-biphenyl]-1,3(2H)-dicarbonitrile (3i): Yield, 75% (76.0 mg); colorless solid; melting point, 140-142 °C; ^{1}H NMR (400 MHz, $CDCl_3$) δ 7.36 (t, J = 8.0 Hz, 1H), 7.07 (ddd, J = 7.8, 1.9, 0.8 Hz, 1H), 7.03 (t, J = 2.1 Hz, 1H), 6.92 (ddd, J = 8.3, 2.5, 0.8 Hz, 1H), 4.52 (s, 2H), 3.86 (s, 3H), 2.85 – 2.69 (m, 3H), 2.43 – 2.27 (m, 2H), 2.21 (ddd, J = 13.5, 11.2, 5.6 Hz, 1H). ^{13}C NMR (101 MHz, $CDCl_3$) δ 160.10 (s), 154.74 (s), 140.02 (s), 130.30 (s), 121.52 (s), 119.09 (s), 117.73 (s), 113.50 (s), 112.03 (s), 71.72 (s), 55.42 (s), 40.92 (s), 35.97 (s), 31.35 (s), 26.39 (s). HR-MS (ESI-TOF) m/z $[M+Na]^+$ calcd. for $C_{15}H_{15}N_3ONa$, 276.1112; found, 276.1107. IR (neat) 3547, 2924, 2853, 2236, 2186, 1639, 1491, 1417, 1262, 848 cm^{-1}.

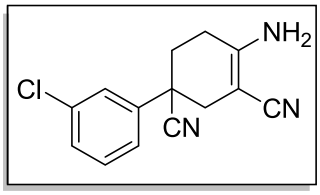

4-amino-3'-chloro-5,6-dihydro-[1,1'-biphenyl]-1,3(2H)-dicarbonitrile (3j): Yield, 58% (59.7 mg); colorless solid; melting point, 155-157 °C; ^{1}H NMR (400 MHz, $CDCl_3$) δ 7.48 (dd, J = 2.9, 1.4 Hz, 1H), 7.43 – 7.36 (m, 3H), 4.57 (s, 2H), 2.75 (ddd, J = 22.7, 20.6, 8.6 Hz, 3H), 2.45 – 2.19 (m, 3H). ^{13}C NMR (101 MHz, $CDCl_3$) δ 154.73 (s), 140.44 (s), 135.23 (s), 130.54 (s), 128.92 (s), 125.97 (s), 123.95 (s), 121.02 (s), 118.95 (s), 71.32 (s), 40.81 (s), 35.92 (s), 31.29 (s), 26.30 (s). HR-MS (ESI-TOF) m/z $[M+Na]^+$ calcd. for $C_{14}H_{12}ClN_3Na$, 280.0617; found, 280.0613. IR (neat) 3454, 3390, 2925, 2853, 2237, 2186, 1639, 1414, 1089, 876 cm^{-1}.

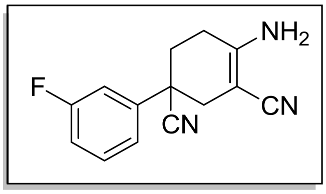

4-amino-3'-fluoro-5,6-dihydro-[1,1'-biphenyl]-1,3(2H)-dicarbonitrile (3k): Yield, 35% (33.7 mg); colorless solid; melting point, 134-136 °C; ^{1}H NMR (400 MHz, $CDCl_3$) δ 7.43 (td, J = 8.0, 6.1 Hz, 1H), 7.34 – 7.26 (m, 1H), 7.23 – 7.16 (m, 1H), 7.09 (td, J = 8.3, 1.9 Hz, 1H), 4.60 (s, 2H), 2.86 – 2.65 (m, 3H), 2.46 – 2.26 (m, 2H), 2.25 – 2.14 (m, 1H). ^{13}C NMR (101 MHz, $CDCl_3$) δ 162.99 (d, J = 247.6 Hz), 154.88 (s), 140.94 (d, J = 7.2 Hz), 130.92 (d, J = 8.2 Hz), 121.42 (d, J = 3.2 Hz), 121.15 (s), 119.06 (s), 115.71 (d, J = 20.9 Hz), 113.07 (d, J = 23.4 Hz), 71.15 (s), 40.82 (s), 35.91 (s), 31.32 (s), 26.29 (s). HR-MS (ESI-TOF) m/z $[M+Na]^+$ calcd. for $C_{14}H_{12}FN_3Na$, 264.0912; found, 264.0907. IR (neat) 3439, 2925, 2852, 2342, 2240, 2184, 1640, 1563, 1413, 1099, 814 cm^{-1}.

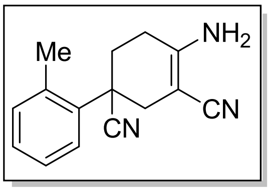

4-amino-2'-methyl-5,6-dihydro-[1,1'-biphenyl]-1,3(2H)-dicarbonitrile (3l): Yield, 51% (48.4 mg); colorless solid; melting point, 135-137 °C; ^{1}H NMR (400 MHz, $CDCl_3$) δ 7.32 – 7.28 (m, 2H), 7.25 (dd, J = 4.0, 2.4 Hz, 2H), 4.54 (s, 2H), 3.06 (dd, J = 15.6, 0.9 Hz, 1H), 2.81 – 2.71 (m, 1H), 2.69 (s, 3H), 2.66 – 2.60 (m, 1H), 2.48 – 2.41 (m, 1H), 2.40 – 2.36 (m, 1H), 2.36 – 2.31 (m, 1H). ^{13}C NMR (101 MHz, $CDCl_3$) δ 154.93 (s), 136.64 (s), 135.23 (s), 133.13 (s), 128.58 (s), 126.71 (s), 125.05 (s), 121.23 (s), 99.98 (s), 71.23 (s), 38.48 (s), 34.32 (s), 30.35 (s), 26.15 (s), 21.22 (s). HR-MS (ESI-TOF) m/z $[M+Na]^+$ calcd. for $C_{15}H_{15}N_3Na$, 260.1163; found, 260.1158. IR (neat) 3453, 3364, 2925, 2854, 2232, 2185, 1639, 1413, 1194, 1021, 929 cm^{-1}.

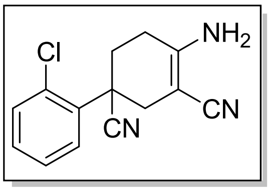

4-amino-2'-chloro-5,6-dihydro-[1,1'-biphenyl]-1,3(2H)-dicarbonitrile (3m): Yield, 34% (35.0 mg); colorless solid; melting point, 154-156 °C; ^{1}H NMR (400 MHz, $CDCl_3$) δ 7.53 – 7.47 (m, 1H), 7.42 (dt, J = 7.4, 3.2 Hz, 1H), 7.38 – 7.33 (m, 2H), 4.56 (s, 2H), 3.22 (d, J = 15.2 Hz, 1H), 2.84 – 2.67 (m, 2H), 2.57 – 2.43 (m, 2H), 2.32 (dt, J = 9.0, 4.8 Hz, 1H). ^{13}C NMR (101 MHz, $CDCl_3$) δ 154.85 (s), 134.11 (s), 133.55 (s), 132.22 (s), 130.07 (s), 127.62 (s), 127.40 (s), 120.16 (s), 119.18 (s), 70.96 (s), 39.30 (s), 33.26 (s), 29.40 (s), 25.99 (s). HR-MS (ESI-TOF) m/z $[M+Na]^+$ calcd. for $C_{14}H_{12}ClN_3Na$, 280.0617; found, 280.0611. IR (neat) 3442, 3363, 2924, 2854, 2234, 2185, 1640, 1414, 1040, 840 cm^{-1}.

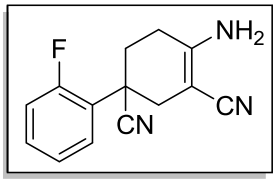

4-amino-2'-fluoro-5,6-dihydro-[1,1'-biphenyl]-1,3(2H)-dicarbonitrile (3n): Yield, 16% (15.4 mg); colorless solid; melting point, 137-139 °C; ^{1}H NMR (400 MHz, $CDCl_3$) δ 7.48 (td, J = 7.9, 1.6 Hz, 1H), 7.44 – 7.36 (m, 1H), 7.26 – 7.12 (m, 2H), 4.56 (s, 2H), 2.96 – 2.82 (m, 2H), 2.80 – 2.68 (m, 1H), 2.49 – 2.40 (m, 1H), 2.40 – 2.30 (m, 2H). ^{13}C NMR (101 MHz, $CDCl_3$) δ 160.59 (d, J = 248.0 Hz), 154.80 (s), 130.73 (d, J = 8.9 Hz), 127.45 (d, J = 3.3 Hz), 124.98 (d, J = 10.6 Hz), 124.85 (d, J = 3.5 Hz), 120.49 (s), 119.15 (s), 117.09 (d, J = 22.5 Hz), 71.11 (s), 38.32 (s), 33.41 (d, J = 2.7 Hz), 29.38 (d, J = 4.2 Hz), 26.07 (s). HR-MS (ESI-TOF) m/z $[M+Na]^+$ calcd. for $C_{14}H_{12}FN_3Na$, 264.0912; found, 264.0910. IR (neat) 3450, 3364, 2925, 2854, 2233, 2185, 1642, 1412, 1048, 837 cm^{-1}.

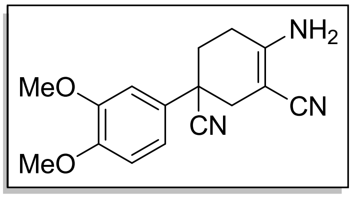

4-amino-3',4'-dimethoxy-5,6-dihydro-[1,1'-biphenyl]-1,3(2H)-dicarbonitrile (3o): Yield, 73% (82.7 mg); colorless solid; melting point, 165-167 °C; ^{1}H NMR (400 MHz, $CDCl_3$) δ 7.03 – 6.99 (m, 2H), 6.91 – 6.88 (m, 1H), 4.52 (s, 2H), 3.94 (s, 3H), 3.92 (s, 3H), 2.85 – 2.70 (m, 3H), 2.41 – 2.29 (m, 2H), 2.24 – 2.18 (m, 1H). ^{13}C NMR (101 MHz, $CDCl_3$) δ 154.71 (s), 149.29 (s), 149.08 (s), 130.80 (s), 121.76 (s), 119.08 (s), 117.62 (s), 111.24 (s), 108.95 (s), 71.72 (s), 56.07 (s), 56.02 (s), 40.46 (s), 36.07 (s), 31.62 (s), 26.44 (s). HR-MS (ESI-TOF) m/z $[M+Na]^+$ calcd. for $C_{16}H_{17}N_3O_2Na$, 306.1218; found, 306.1215. IR (neat) 3445, 2924, 2852, 2185, 1640, 1520, 1264, 1520, 1264, 1148, 1021, 807 cm^{-1}.

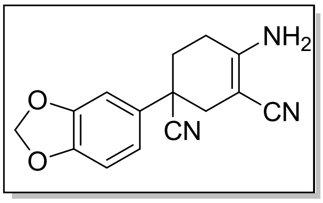

4-amino-1-(benzo[d][1,3]dioxol-5-yl)cyclohex-3-ene-1,3-dicarbonitrile (3p): Yield, 78% (83.4 mg); colorless solid; melting point, 199-201 °C; ^{1}H NMR (400 MHz, $CDCl_3$) δ 6.98 (dd, J = 8.1, 2.1 Hz, 1H), 6.95 (d, J = 1.8 Hz, 1H), 6.85 (d, J = 8.1 Hz, 1H), 6.03 (s, 2H), 4.51 (s, 2H), 2.83 – 2.65 (m, 3H), 2.41 – 2.25 (m, 2H), 2.16 (ddd, J = 13.4, 11.0, 5.6 Hz, 1H). ^{13}C NMR (101 MHz, $CDCl_3$) δ 154.69 (s), 148.41 (s), 147.73 (s), 132.19 (s), 119.11 (s), 108.61 (s), 106.23 (s), 101.61 (s), 99.98 (s), 71.67 (s), 40.51 (s), 36.13 (s), 31.58 (s), 26.38 (s). HR-MS (ESI-TOF) m/z $[M+Na]^+$ calcd. for $C_{15}H_{13}N_3O_2Na$, 290.0905; found, 290.0902. IR (neat) 3451, 2924, 2853, 2238, 2183, 1640, 1562, 1414, 1248, 811 cm^{-1}.

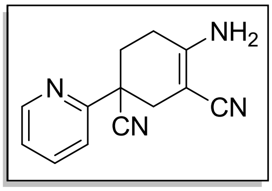

4-amino-1-(pyridin-2-yl)cyclohex-3-ene-1,3-dicarbonitrile (3q): Yield, 83% (74.4 mg); colorless solid; melting point, 170-172 °C; ^{1}H NMR (400 MHz, $CDCl_3$) δ 8.68 – 8.62 (m), 7.81 (ddd, J = 7.2, 5.3, 1.8 Hz, 1H), 7.50 (d, J = 7.8 Hz, 1H), 7.35 (ddd, J = 7.6, 4.9, 0.9 Hz, 1H), 4.50 (s, 1H), 4.19 (t, J = 7.0 Hz, 1H), 2.69 – 2.34 (m, 6H). ^{13}C NMR (101 MHz, $CDCl_3$) δ 152.74 (s), 150.29 (s), 137.77 (s), 123.74 (s), 122.26 (s), 120.84 (s), 118.51 (s), 38.06 (s), 34.40 (s), 30.49 (s), 29.18 (s), 14.98 (s). HR-MS (ESI-TOF) m/z $[M+Na]^+$ calcd. for $C_{13}H_{12}N_4Na$, 247.0959; found, 247.0956. IR (neat) 3443, 2925, 2853, 2248, 2186, 1639, 1589, 1416, 1020, 785 cm^{-1}.

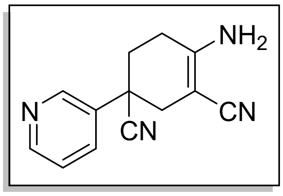

4-amino-1-(pyridin-3-yl)cyclohex-3-ene-1,3-dicarbonitrile (3r): Yield, 80% (71.7 mg); colorless solid; melting point, 174-176 °C; ^{1}H NMR (400 MHz, $CDCl_3$) δ 8.79 (d, J = 1.7 Hz, 1H), 8.67 (d, J = 2.3 Hz, 1H), 7.87 (ddd, J = 6.1, 1.9, 1.2 Hz, 1H), 7.47 – 7.37 (m, 1H), 4.58 (s, 2H), 2.90 – 2.73 (m, 4H), 2.71 – 2.49 (m, 2H). ^{13}C NMR (101 MHz, $CDCl_3$) δ 154.56 (s), 149.85 (s), 148.53 (s), 134.91 (s), 133.86 (s), 123.94 (s), 120.49 (s), 118.75 (s), 71.29 (s), 39.50 (s), 35.78 (s), 31.23 (s), 26.17 (s). HR-MS (ESI-TOF) m/z $[M+Na]^+$ calcd. for $C_{13}H_{12}N_4Na$, 247.0959; found, 247.0955. IR (neat) 3440, 2924, 2853, 2247, 2185, 1691, 1639, 1420, 1192, 812 cm^{-1}.

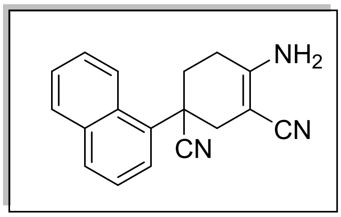

4-amino-1-(naphthalen-2-yl)cyclohex-3-ene-1,3-dicarbonitrile (3s): Yield, 79% (86.4 mg); colorless solid; melting point, 220-222 °C; ^{1}H NMR (400 MHz, $CDCl_3$) δ 8.53 (d, J = 8.6 Hz, 1H), 7.95 (t, J = 6.5 Hz, 1H), 7.93 – 7.88 (m, 1H), 7.67 (ddd, J = 8.6, 6.9, 1.5 Hz, 1H), 7.62 – 7.56 (m, 1H), 7.52 – 7.46 (m, 2H), 4.56 (s, 2H), 3.41 – 3.30 (m, 1H), 2.89 – 2.75 (m, 2H), 2.73 – 2.62 (m, 1H), 2.51 (ddd, J = 13.1, 10.4, 5.3 Hz, H), 2.41 – 2.30 (m, 1H). ^{13}C NMR (101 MHz, $CDCl_3$) δ 154.88 (s), 134.70 (s), 132.77 (s), 130.20 (s), 130.05 (s), 129.78 (s), 126.75 (s), 126.09 (s), 125.03 (s), 124.04 (s), 123.41 (s), 121.79 (s), 119.22 (s), 71.32 (s), 38.23 (s), 35.21 (s), 31.09 (s), 26.17 (s). HR-MS (ESI-TOF) m/z $[M+Na]^+$ calcd. for $C_{18}H_{15}N_3Na$, 296.1163; found, 296.1159. IR (neat) 3455, 2923, 2853, 2239, 2183, 1639, 1560, 1413, 1270, 802 cm^{-1}.

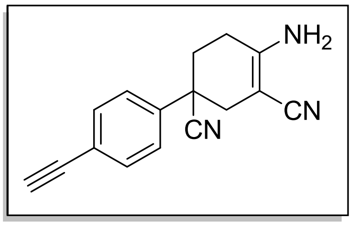

4-amino-4'-ethynyl-5,6-dihydro-[1,1'-biphenyl]-1,3(2H)-dicarbonitrile (3t): Yield, 54% (53.4 mg); colorless solid; melting point, 212-214 °C; ^{1}H NMR (400 MHz, $CDCl_3$) δ 7.58 (d, J = 1.9 Hz, 1H), 7.56 – 7.55 (m, 1H), 7.48 – 7.46 (m, 1H), 7.46 – 7.44 (m, 1H), 4.53 (s, 2H), 3.15 (d, J = 1.3 Hz, 1H), 2.85 – 2.69 (m, 3H), 2.42 – 2.27 (m, 2H), 2.22 (ddd, J = 13.5, 11.0, 5.6 Hz, 1H). ^{13}C NMR (101 MHz, $CDCl_3$) δ 156.17 (s), 154.64 (s), 138.94 (s), 132.92 (s), 125.70 (s), 122.68 (s), 118.93 (s), 82.52 (s), 78.52 (s), 71.56 (s), 40.88 (s), 35.75 (s), 31.35 (s), 26.28 (s). HR-MS (ESI-TOF) m/z $[M+Na]^+$ calcd. for $C_{16}H_{13}N_3Na$, 270.1007; found, 270.1001. IR (neat) 3455, 3362, 3235, 2924, 2853, 2242, 2183, 1643, 1561, 1413, 1020, 831 cm^{-1}.

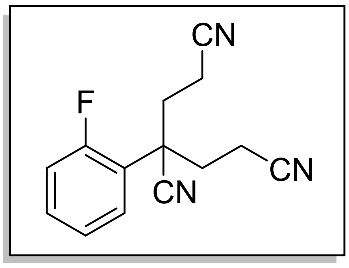

3-(2-fluorophenyl)pentane-1,3,5-tricarbonitrile (3n'): Yield, 36% (34.7 mg); colorless solid; melting point, 85-87 °C; ^{1}H NMR (400 MHz, $CDCl_3$) δ 7.61 – 7.53 (m, 1H), 7.51 – 7.43 (m, 1H), 7.27 (dt, J = 7.6, 1.5 Hz, 1H), 7.22 – 7.13 (m, 1H), 2.70 – 2.59 (m, 2H), 2.56 – 2.37 (m, 4H), 2.26 – 2.15 (m, 2H). ^{13}C NMR (101 MHz, $CDCl_3$) δ 159.84 (d, J = 246.9 Hz), 132.16 (d, J = 9.0 Hz), 130.13 (d, J = 3.2 Hz), 125.57 (d, J = 3.5 Hz), 119.88 (d, J = 10.3 Hz), 119.06 (s), 117.68 (d, J = 22.3 Hz), 117.65 (s),

46.69 (s), 46.65 (s), 33.57 (s), 33.52 (s), 14.04 (s). HR-MS (ESI-TOF) m/z $[M+Na]^+$ calcd. for $C_{14}H_{12}FN_3Na$, 264.0907; found 264.0900. IR (neat) 3434, 3333, 3186, 2256, 2270, 2170, 1639, 1400, 1259, 926 cm^{-1}.

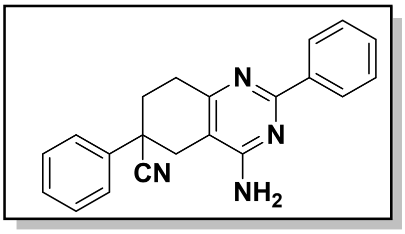

4-amino-2,6-diphenyl-5,6,7,8-tetrahydroquinazoline-6-carbonitrile (5a): Yield, 52% (33.9 mg); colorless solid; melting point, 180-182 °C; ^1H NMR (400 MHz, $CDCl_3$) δ 8.35 – 8.30 (m, 2H), 7.53 – 7.36 (m, 8H), 5.20 (s, 2H), 3.33 – 3.20 (m, 1H), 3.05 – 2.92 (m, 2H), 2.86 (d, J = 16.3 Hz, 1H), 2.48 – 2.30 (m, 2H). ^{13}C NMR (101 MHz, $CDCl_3$) δ 162.3 (s), 161.5 (s), 139.1 (s), 138.0 (s), 130.2 (s), 129.3 (s), 128.6 (s), 128.4 (s), 128.1 (s), 125.6 (s), 122.1 (s), 106.3 (s), 41.3 (s), 35.0 (s), 32.3 (s), 29.5 (s). HR-MS (ESI-TOF) m/z $[M+Na]^+$ calcd. for $C_{21}H_{18}N_4Na$, 349.1429; found, 349.1425. IR (neat) 3508, 3348, 3908, 2360, 2239, 1924. 1618, 1552, 1396, 1008 cm^{-1}.

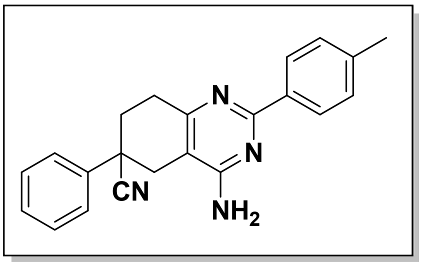

4-amino-6-phenyl-2-(p-tolyl)-5,6,7,8-tetrahydroquinazoline-6-carbonitrile (5b): Yield, 49% (33.3 mg); colorless solid; melting point, 234-236 °C; ^1H NMR (400 MHz, $CDCl_3$) δ 8.21 (d, J = 8.2 Hz, 2H), 7.55 – 7.50 (m, 2H), 7.47 – 7.41 (m, 2H), 7.38 (ddd, J = 7.4, 3.5, 1.2 Hz, 1H), 7.25 (d, J = 8.2 Hz, 2H), 4.94 (s, 2H), 3.33 – 3.21 (m, 1H), 2.98 (ddd, J = 33.9, 24.9, 16.1 Hz, 3H), 2.51 – 2.33 (m, 2H), 2.40 (s, 3H). ^{13}C NMR (101 MHz, $CDCl_3$) δ 162.4 (s), 161.5 (s), 161.3 (s), 140.3 (s), 139.1 (s), 135.2 (s), 129.3 (s), 129.2 (s), 128.6 (s), 127.9 (s), 125.6 (s), 122.0 (s), 105.9 (s), 41.3 (s), 35.1 (s), 32.4 (s), 29.5 (s), 21.5 (s). HR-MS (ESI-TOF) m/z $[M+Na]^+$ calcd. for $C_{22}H_{20}N_4Na$, 363.1585; found, 363.1581. IR (neat) 3499, 3332, 2985, 2341, 2293, 1916, 1612. 1456, 1263, 978 cm^{-1}.

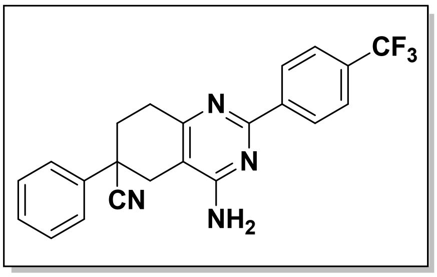

4-amino-6-phenyl-2-(4-(trifluoromethyl)phenyl)-5,6,7,8-tetrahydroquinazoline-6-carbonitrile (5c): Yield, 17% (13.4 mg); colorless solid; melting point, 219-221 °C; ^1H NMR (400 MHz, $CDCl_3$) δ 8.45 (d, J = 8.1 Hz, 2H), 7.69 (d, J = 8.2 Hz, 2H), 7.56

– 7.51 (m, 2H), 7.48 – 7.42 (m, 2H), 7.42 – 7.36 (m, 1H), 4.98 (s, 2H), 3.36 – 3.24 (m, 1H), 3.12 – 2.91 (m, 3H), 2.54 – 2.37 (m, 2H). ^{13}C NMR (101 MHz, $CDCl_3$) δ 161.54 (d, *J* = 17.0 Hz), 160.81 (s), 141.24 (s), 138.91 (s), 131.57 (q, *J* = 23.0 Hz), 129.23 (s), 128.54 (s), 128.18 (s), 125.45 (s), 125.12 (q, *J* = 4.0 Hz), 124.65 (q, *J* = 258.0 Hz), 121.85 (s), 106.84 (s), 41.10 (s), 34.93 (s), 32.05 (s), 29.31 (s). HR-MS (ESI-TOF) m/z $[M+Na]^+$ calcd. for $C_{22}H_{17}F_3N_4Na$, 417.1303; found, 417.12999. IR (neat) 3510, 3362, 2863, 2382, 2231. 1961. 1613, 1550, 1323, 962 cm^{-1}.

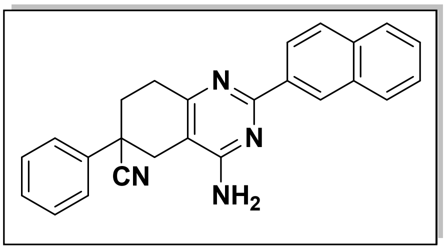

4-amino-2-(naphthalen-2-yl)-6-phenyl-5,6,7,8-tetrahydroquinazoline-6-carbonitrile (5d): Yield, 41% (30.9 mg); colorless solid; melting point, 164-166 °C; ^{1}H NMR (400 MHz, $CDCl_3$) δ 8.87 (s, 1H), 8.46 (dd, J = 8.6, 1.6 Hz, 1H), 8.04 – 7.96 (m, 1H), 7.95 – 7.82 (m, 2H), 7.59 – 7.50 (m, 4H), 7.49 – 7.37 (m, 3H), 5.11 (s, 2H), 3.40 – 3.26 (m, 1H), 3.13 – 3.00 (m, 2H), 2.92 (d, J = 16.3 Hz, 1H), 2.55 – 2.35 (m, 2H). ^{13}C NMR (101 MHz, $CDCl_3$) δ 162.2 (s), 161.6 (s), 161.4 (s), 139.0 (s), 135.2 (s), 134.4 (s), 133.2 (s), 129.2 (s), 129.0 (s), 128.5 (s), 128.0 (s), 127.9 (s), 127.6 (s), 126.7 (s), 126.0 (s), 125.5 (s), 125.2 (s), 121.9 (s), 106.2 (s), 41.1 (s), 34.9 (s), 32.2 (s), 29.5 (s). HR-MS (ESI-TOF) m/z [M+Na]+ calcd. for $C_{25}H_{20}N_4Na$, 399.1585; found, 399.1580. IR (neat) 3499, 3356, 3001, 2295, 2198, 1968, 1603, 1559, 1362, 949 cm^{-1}.

4. X-ray Structure Determinations

Diffraction data were collected on an Oxford Diffraction Supernova dual diffractometer equipped with an Oxford Cryostream 700 low-temperature apparatus. Cu K\α radiation source (λ = 1.54184 Å) was used for the data collection. Single crystals were coated with Paratone-N oil and mounted on a Nylon loop for diffraction. The data reduction and cell refinement were processed using CrysAlisPro software.[1] Structures were solved by direct methods using the SHELXTL program packages.[2] All non-hydrogen atoms were refined anisotropically and hydrogen atoms were added geometrically. Crystal data and refinement details were given in Tables S1. Other refinement details and explanations were included in individual CIF files.

5. Crystallographic data of compounds

Table S1. Crystallographic Data [a] for compound **3a** **5a**.

Compound	**3a**	**5a** (2158132)
formula	$C_{56}H_{52}N_{12}$	$C_{21}H_{18}N_4$
M	893.09	326.39
crystal system	triclinic	triclinic
space group	P-1	P-1
a, Å	11.5840(4)	6.1541(2)
b, Å	13.3019(4)	16.1600(8)
c, Å	16.7992(5)	18.3677(9)
α, deg	67.011(3)	68.234(5)
β, deg	80.092(3)	89.533(4)
γ, deg	77.442(3)	84.560(4)
V, Å^3	2315.02(14)	1688.08(13)
Z	2	4
μ, mm^{-1}	0.617	0.614
independent data	9191	6364
refined parameters	614	493
$R_1{}^b$, $wR_2{}^c$ (I >2σ(I))	0.0387, 0.1094	0.0557, 0.1422
R_1, wR_2 (all data)	0.0488, 0.1153	0.0721, 0.1516

[a]T = 150(2) K, Cu Kα radiation (λ = 1.54184 Å). [b]$R_1 = \sum||F_o| - |F_c|| / \sum|F_o|$. [c]$wR_2 = \{\sum[w(F_o^2 - F_c^2)^2/(F_o^2)^2]\}^{½}$.

6. ^{1}H NMR and ^{13}C NMR spectra of compounds

^{1}H NMR (400M, $CDCl_3$) and ^{13}C (101M, $CDCl_3$) of 4-amino-5,6-dihydro-[1, 1'-biphenyl]-1,3(2H)-dicarbonitrile (3a):

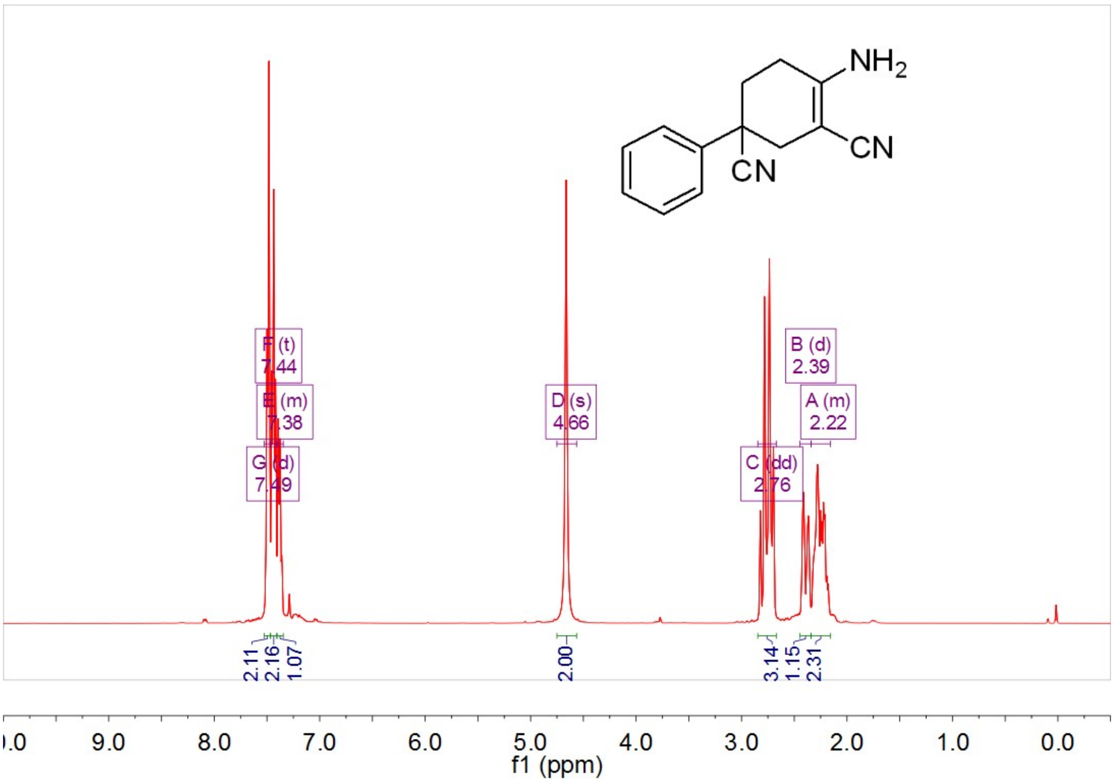

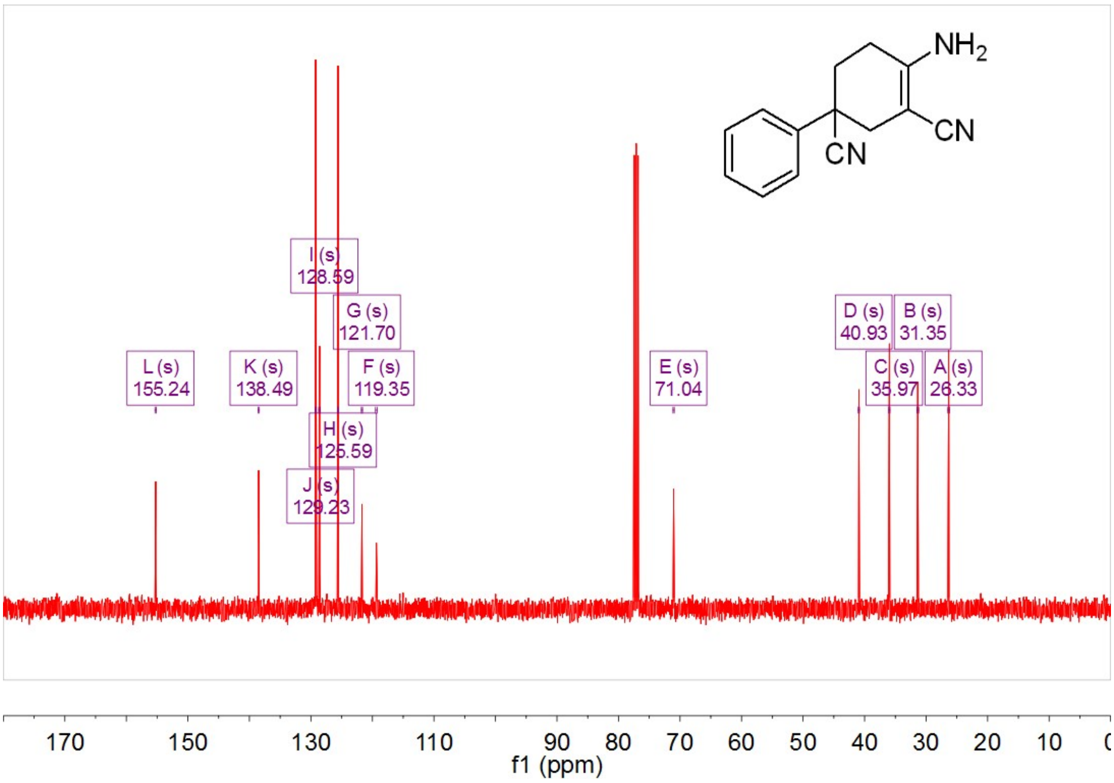

^{1}H NMR (400M, $CDCl_3$) and ^{13}C (101M, $CDCl_3$) of 4-amino-4'-methyl-5,6-dihydro-[1,1'-biphenyl]-1,3(2H)-dicarbonitrile (3b):

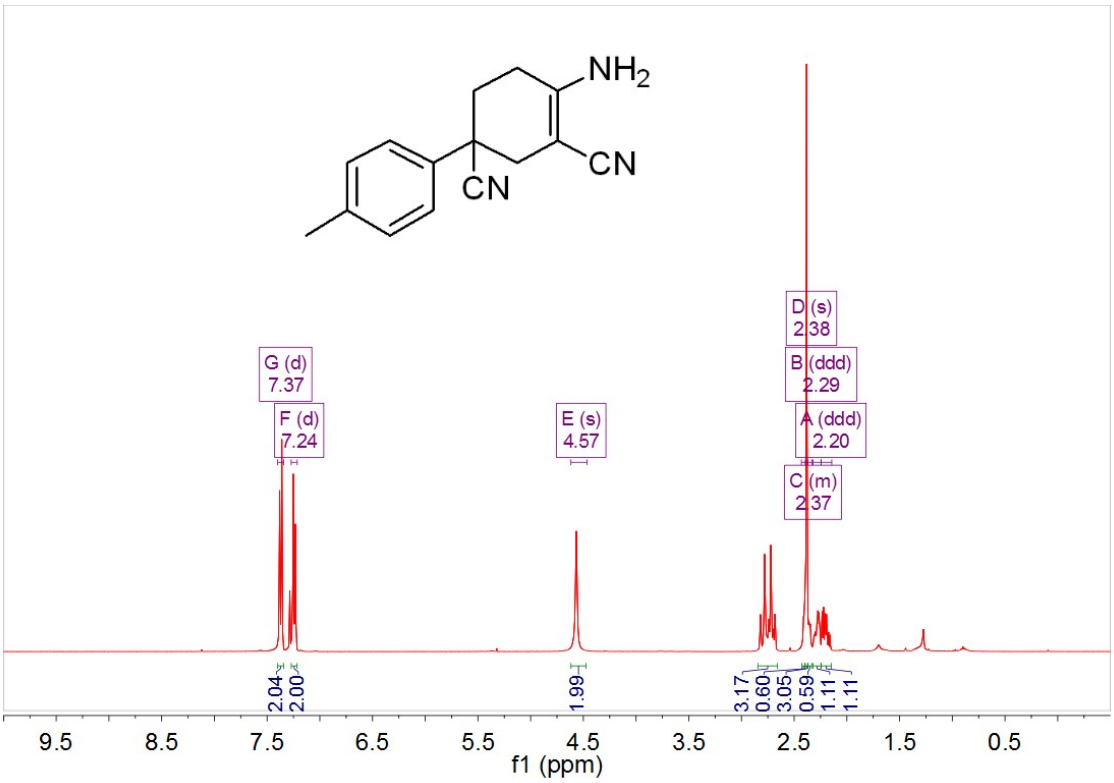

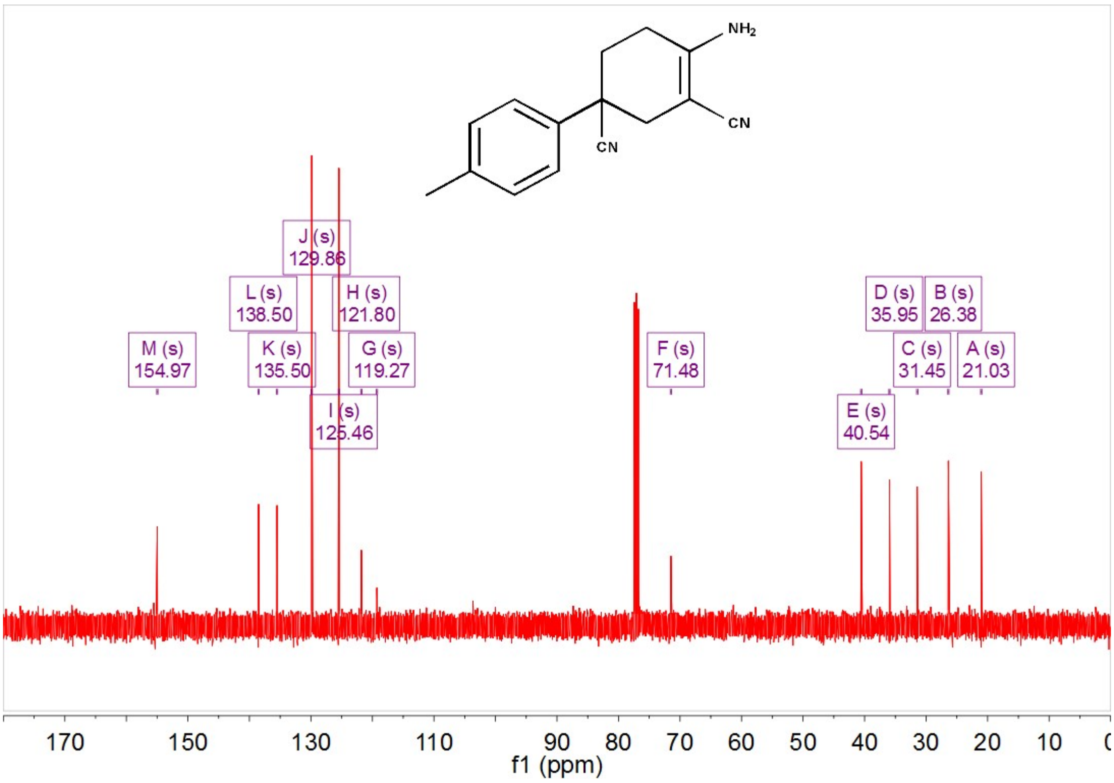

^{1}H NMR (400M, $CDCl_3$) and ^{13}C (101M, $CDCl_3$) of 4-amino-4'-methoxy-5,6 -dihydro-[1,1'-biphenyl]-1,3(2H)-dicarbonitrile (3c):

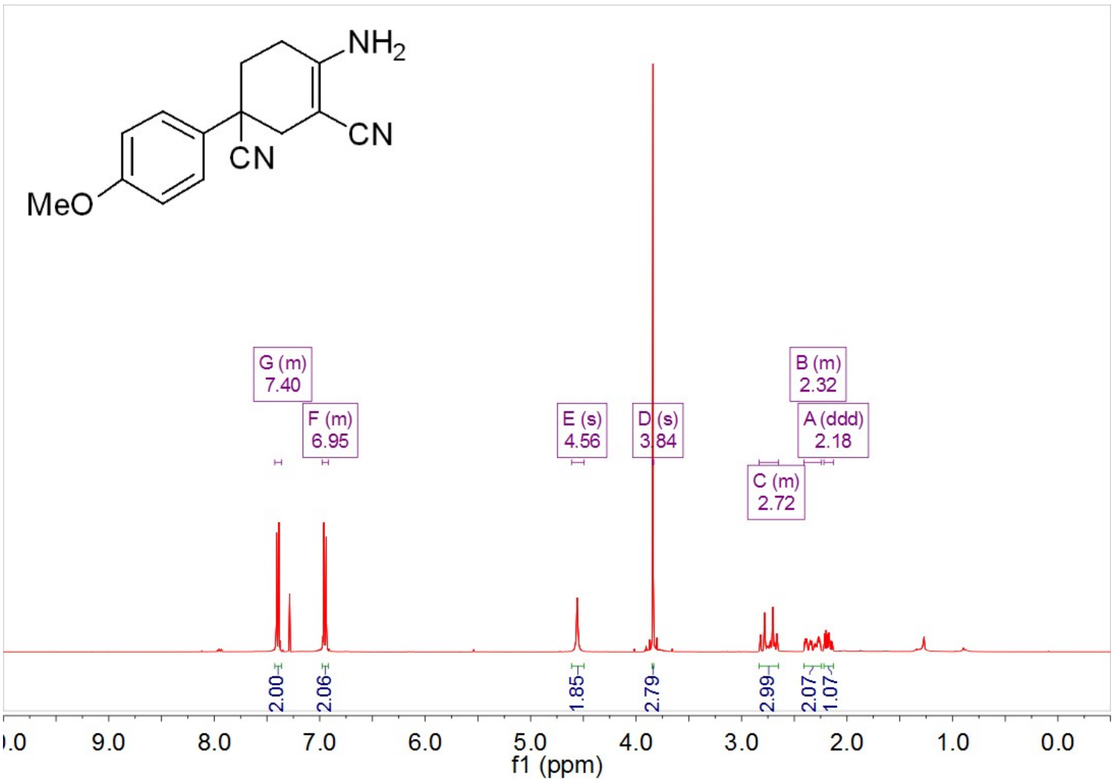

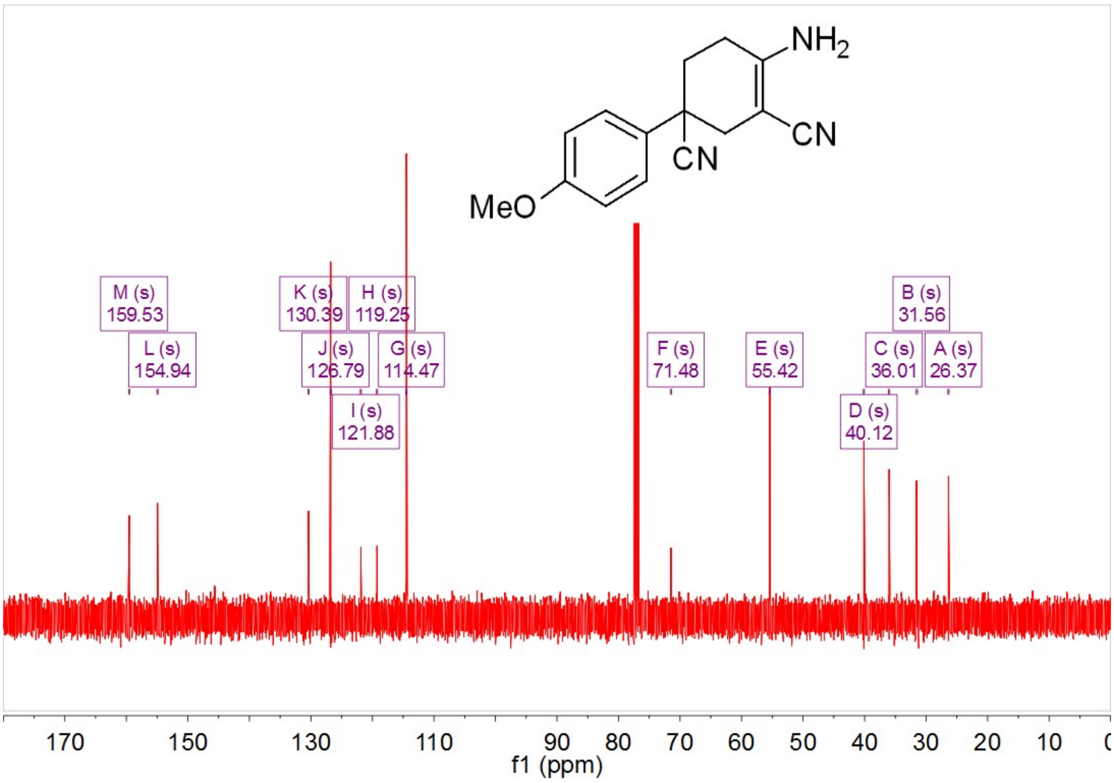

^{1}H NMR (400M, $CDCl_3$) and ^{13}C (101M, $CDCl_3$) of 4-amino-4'-(tert-butyl)-5,6-dihydro-[1,1'-biphenyl]-1,3(2H)-dicarbonitrile (3d):

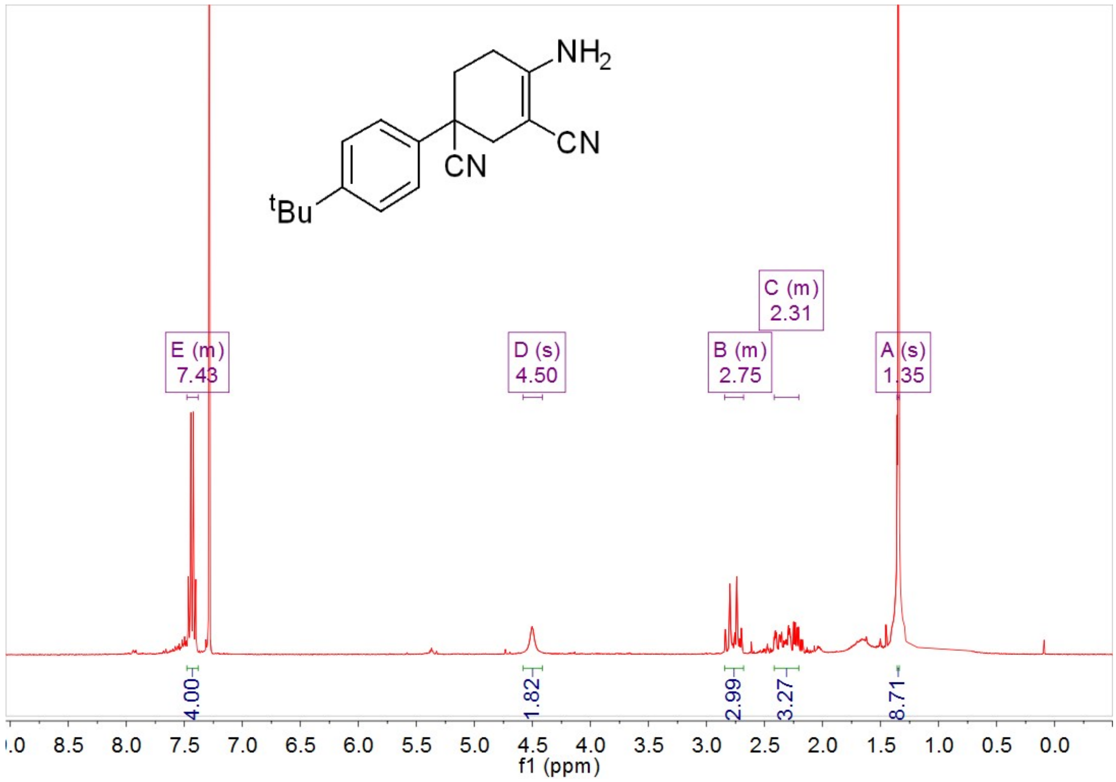

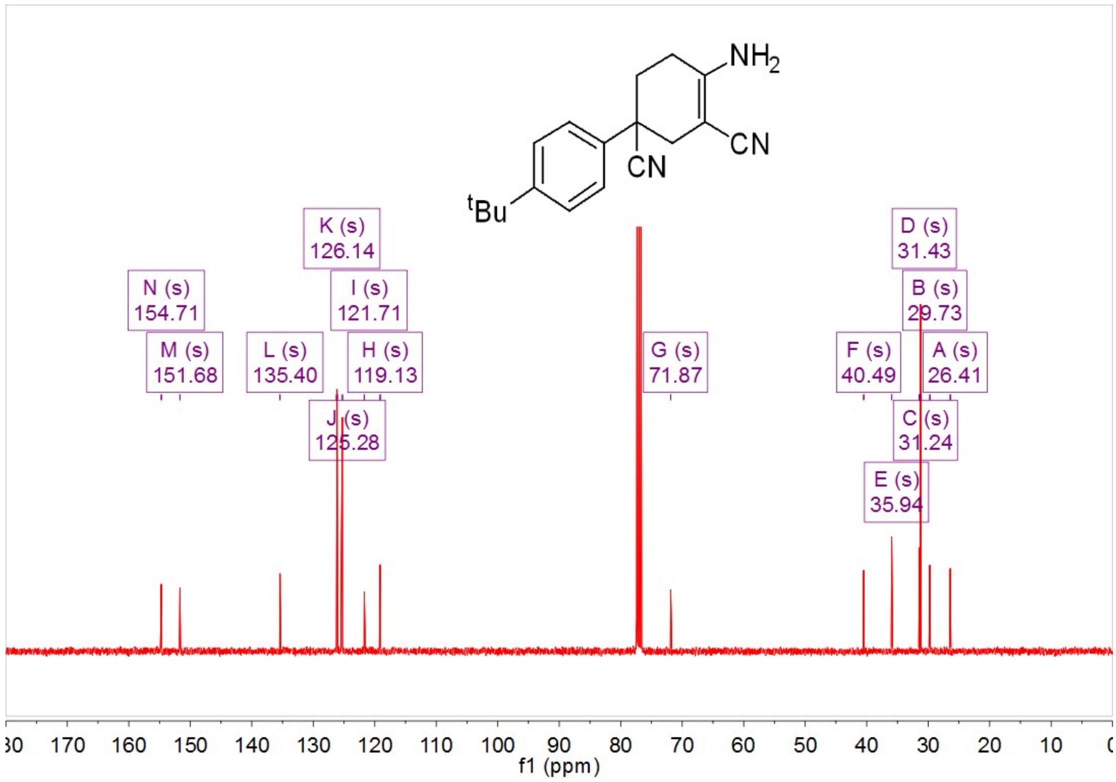

^{1}H NMR (400M, $CDCl_3$) and ^{13}C (101M, $CDCl_3$) of 4-amino-5,6-dihydro-[1, 1':4',1''-terphenyl]-1,3(2H)-dicarbonitrile (3e):

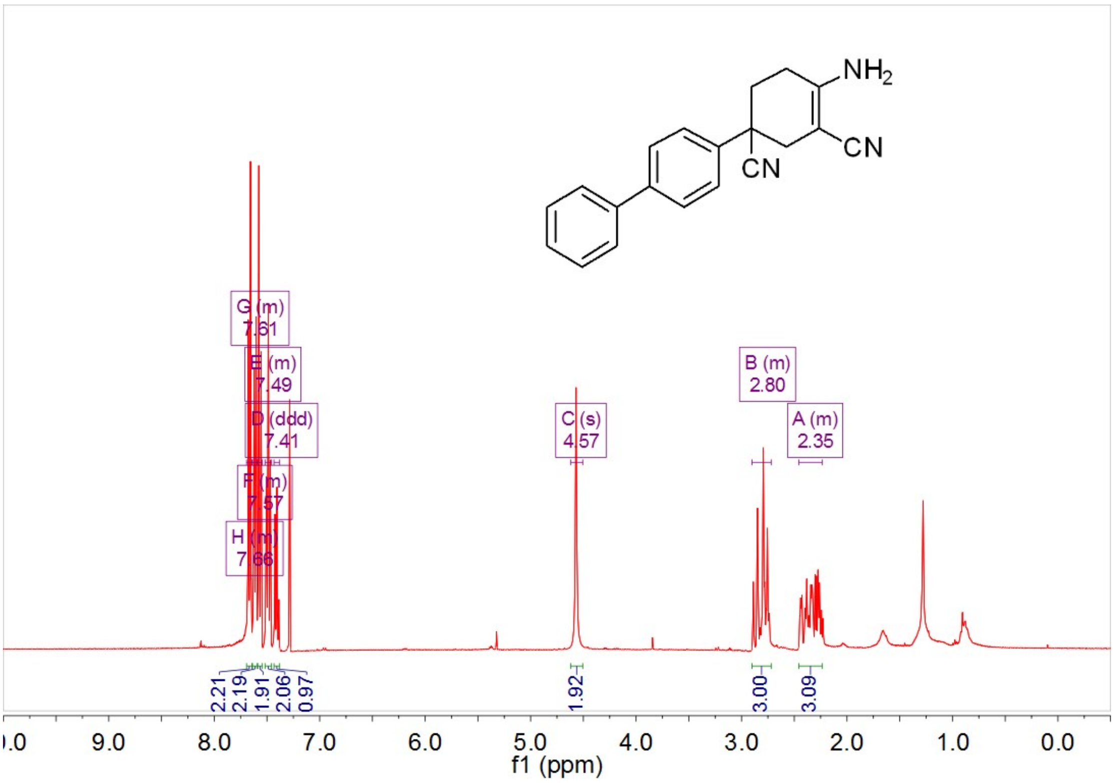

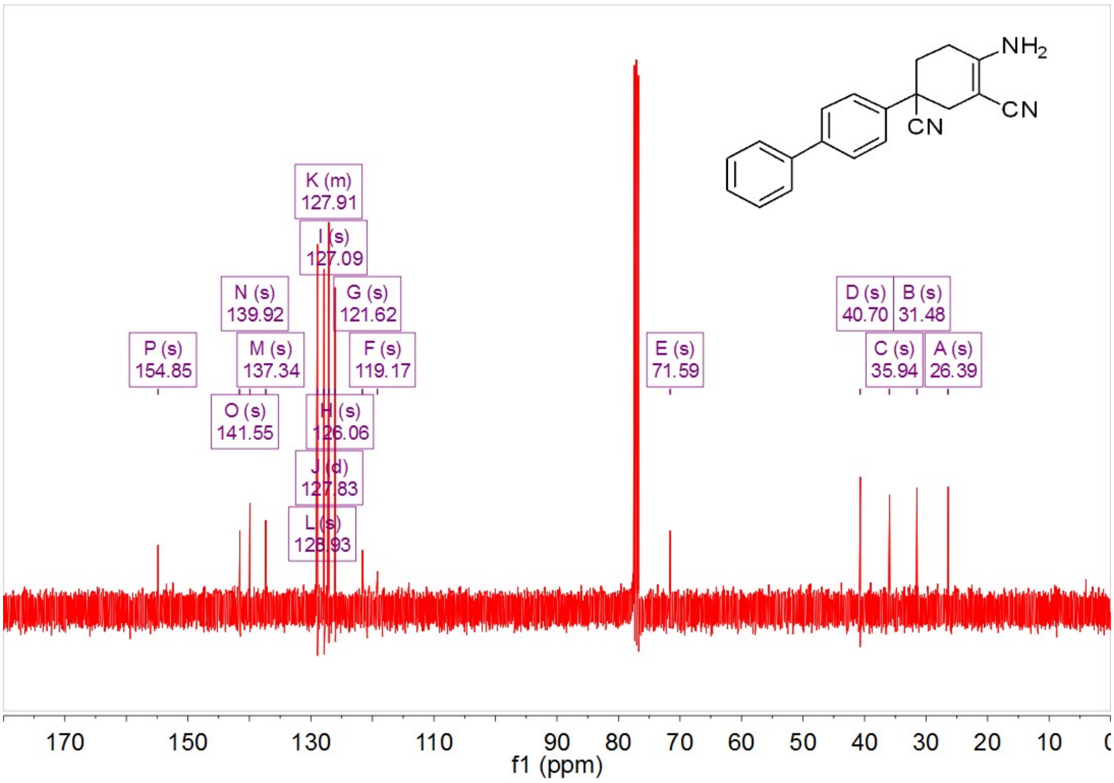

^{1}H NMR (400M, $CDCl_3$) and ^{13}C (101M, $CDCl_3$) of 4-amino-4'-bromo-5,6-dihydro-[1,1'-biphenyl]-1,3(2H)-dicarbonitrile (3f):

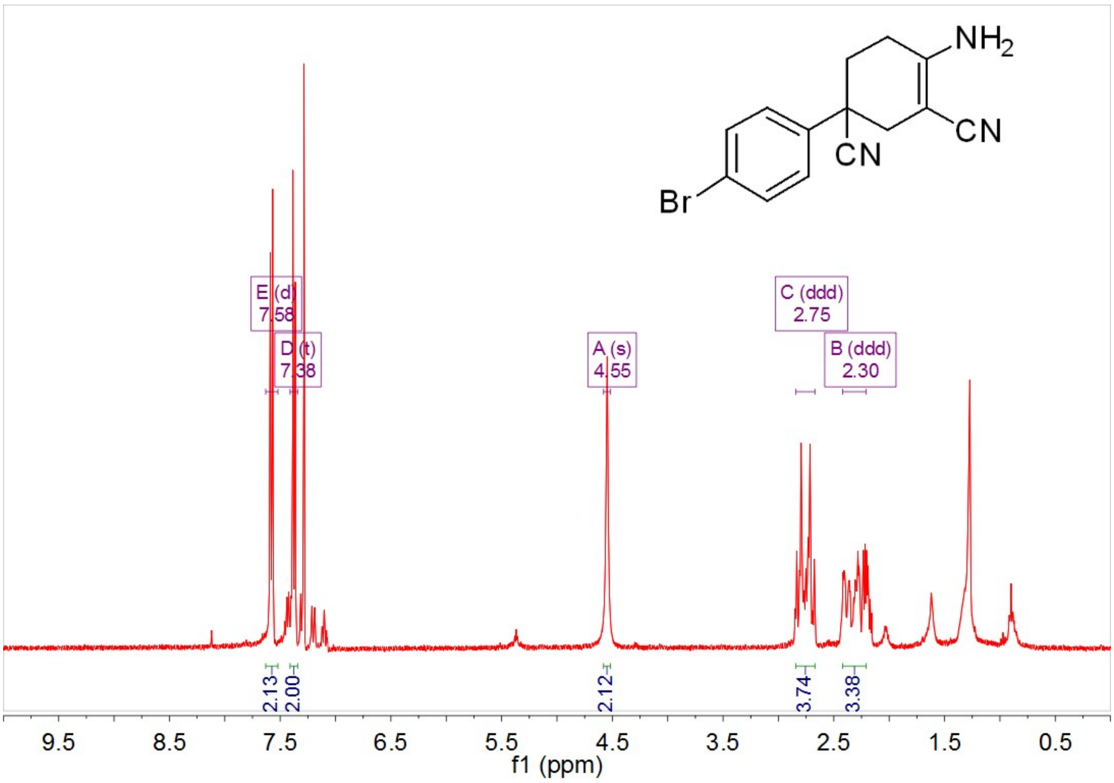

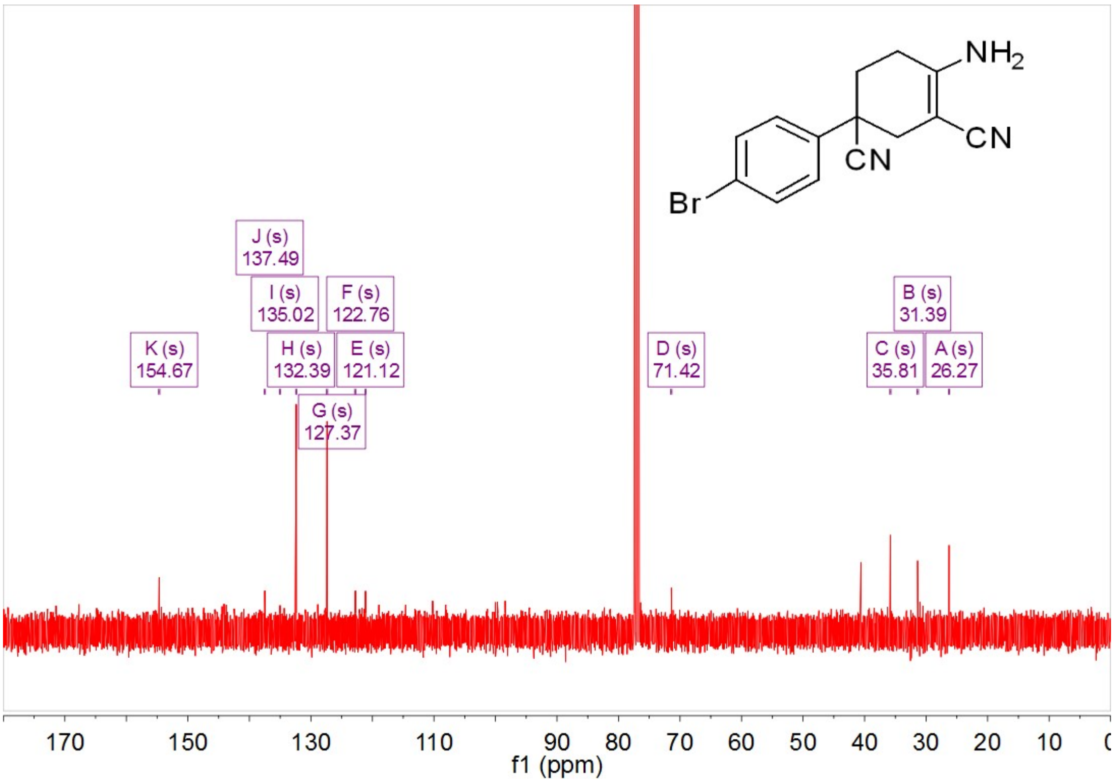

^{1}H NMR (400M, $CDCl_3$) and ^{13}C (101M, $CDCl_3$) of 4-amino-4'-chloro-5,6-dihydro-[1,1'-biphenyl]-1,3(2H)-dicarbonitrile (3g):

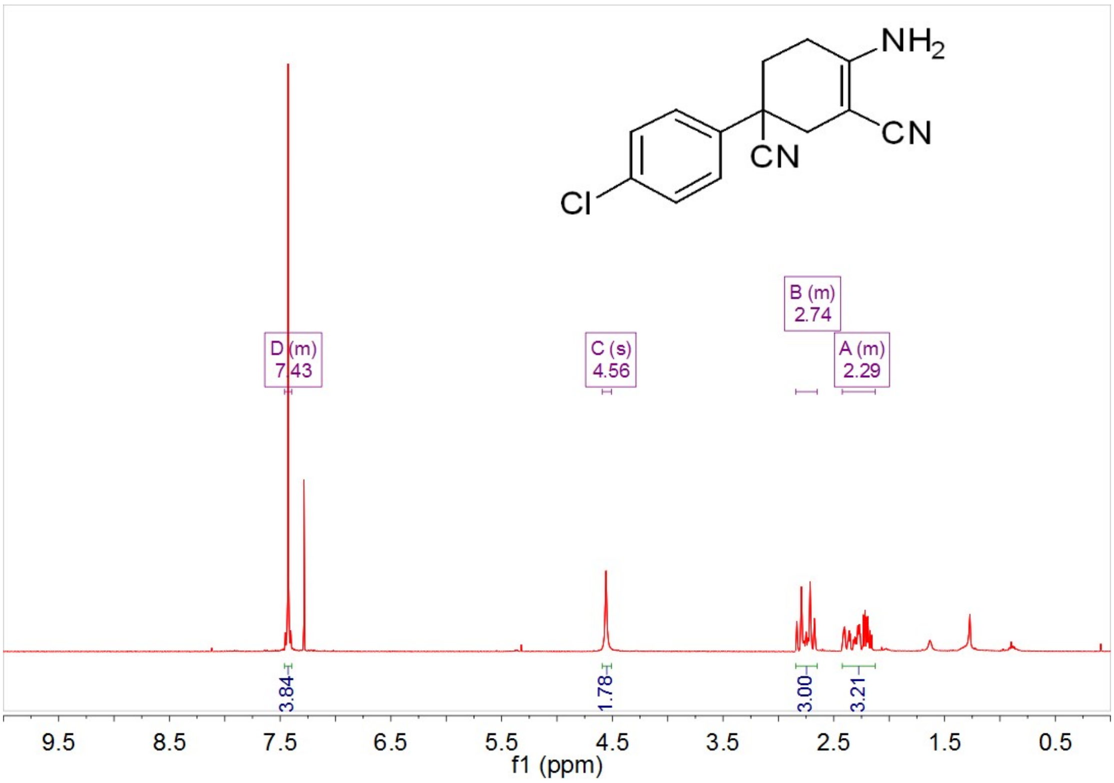

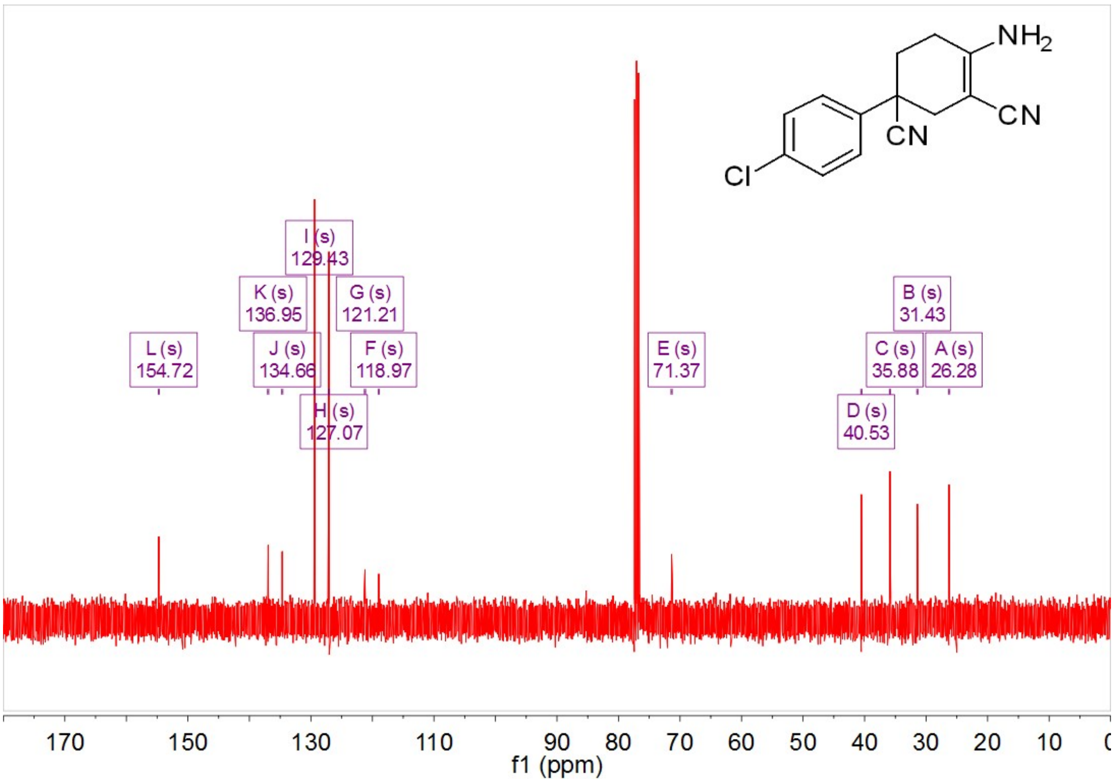

^{1}H NMR (400M, $CDCl_3$) and ^{13}C (101M, $CDCl_3$) of 4-amino-4'-fluoro-5,6-dihydro-[1,1'-biphenyl]-1,3(2H)-dicarbonitrile (3h):

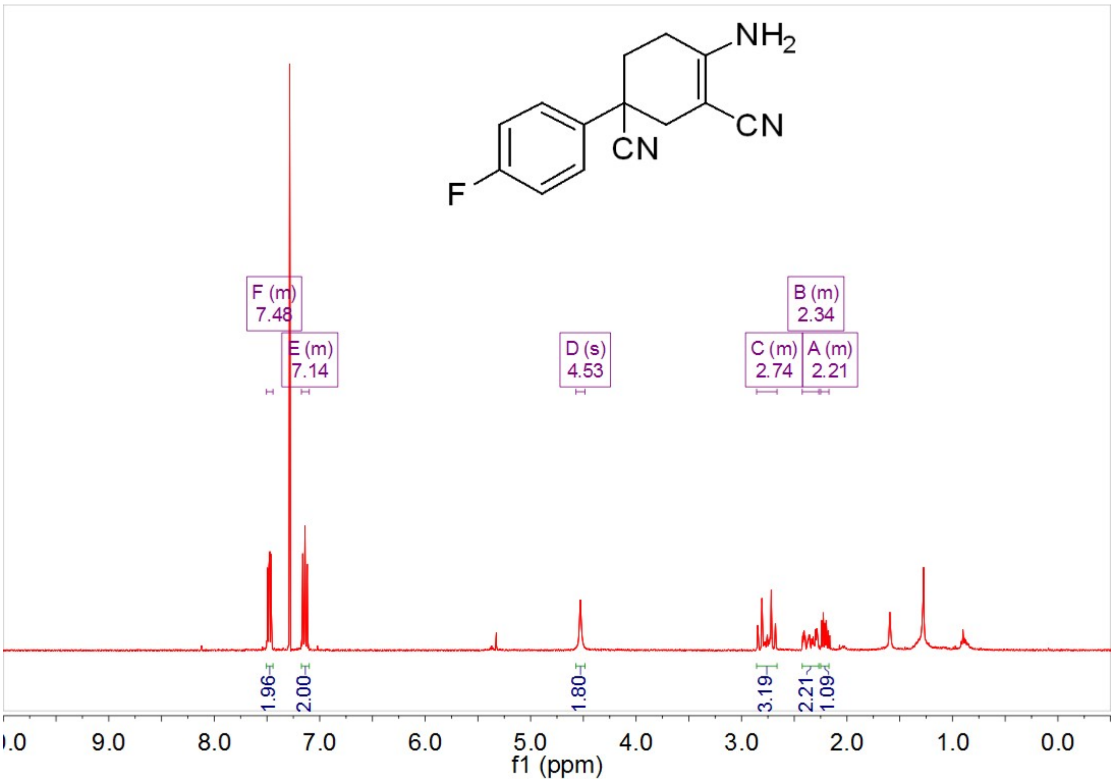

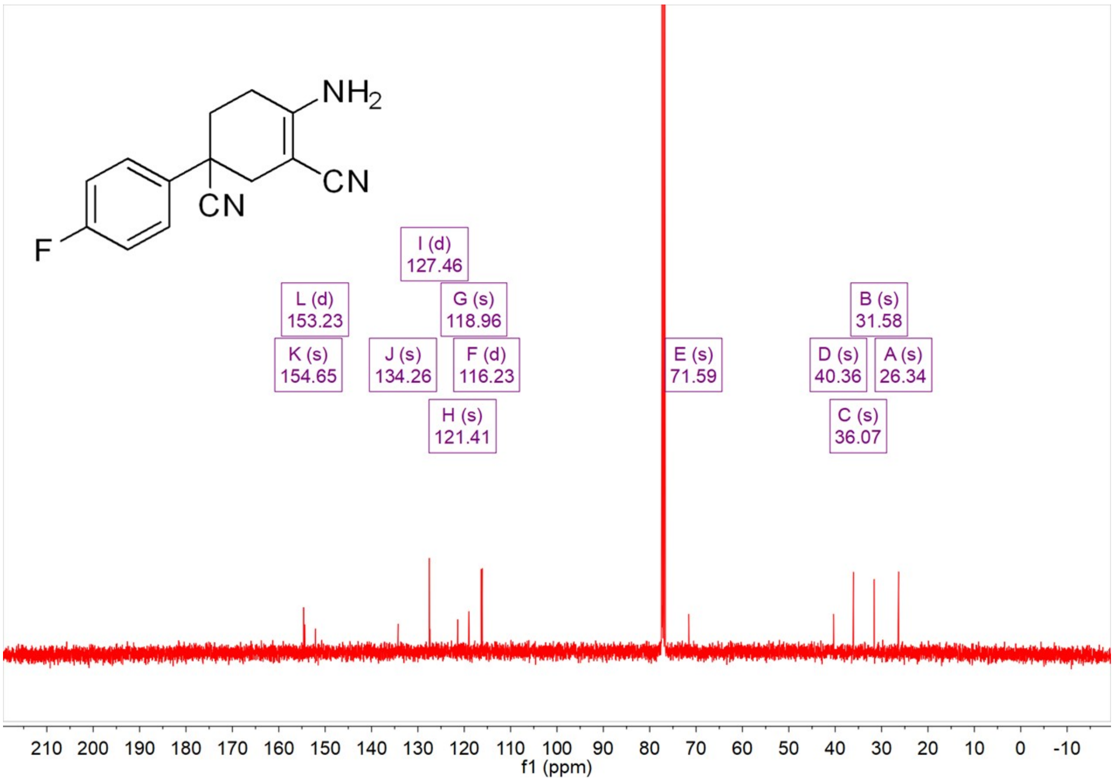

^{1}H NMR (400M, $CDCl_3$) and ^{13}C (101M, $CDCl_3$) of 4-amino-3'-methoxy-5,6-dihydro-[1,1'-biphenyl]-1,3(2H)-dicarbonitrile (3i)

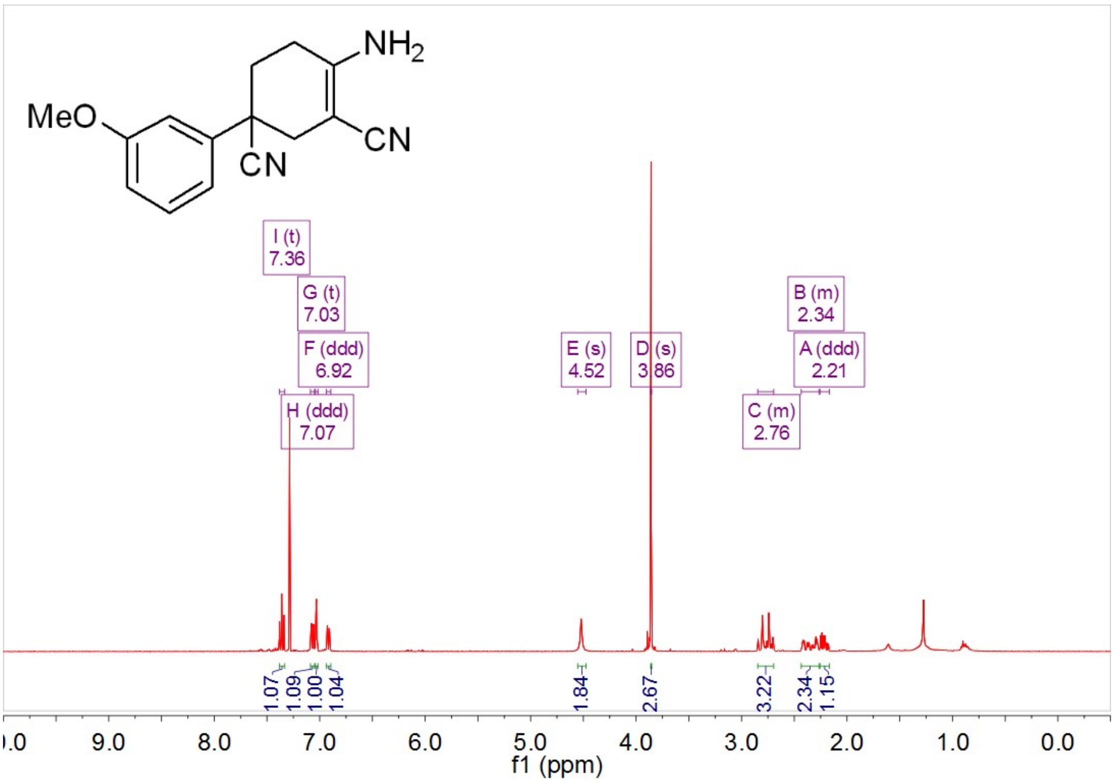

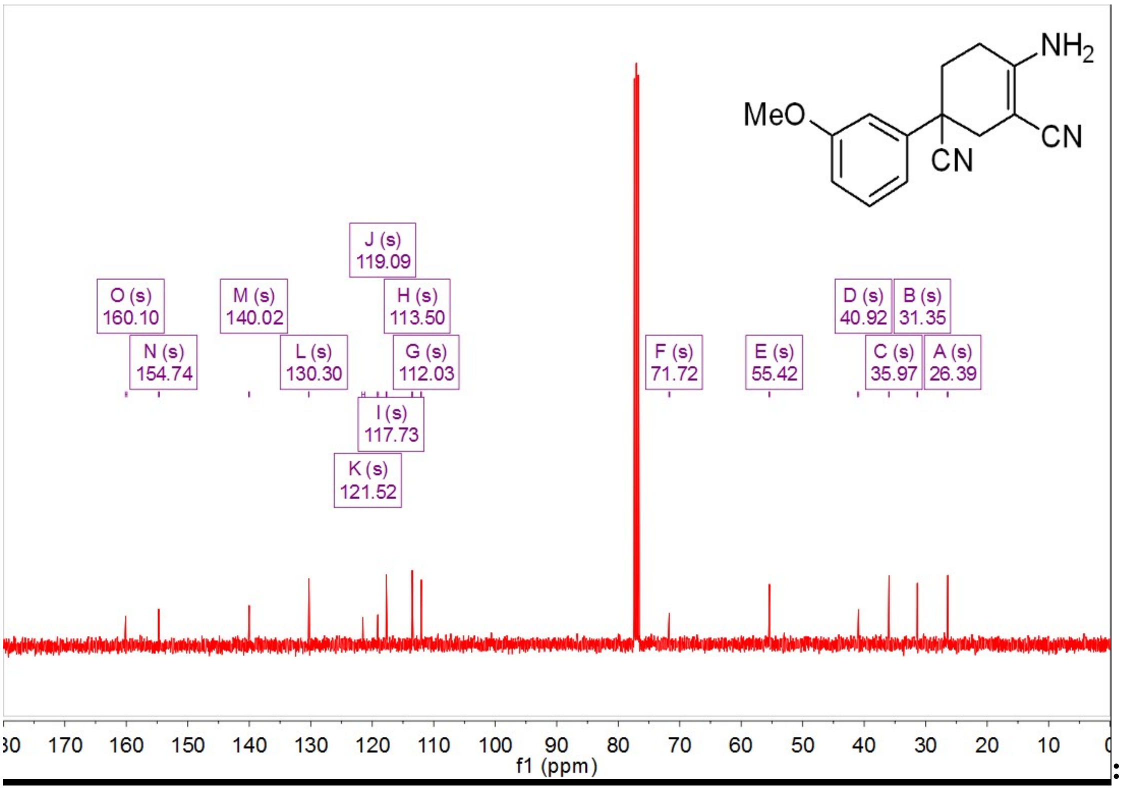

^{1}H NMR (400M, $CDCl_3$) and ^{13}C (101M, $CDCl_3$) of 4-amino-3'-chloro-5,6-d

ihydro-[1,1'-biphenyl]-1,3(2H)-dicarbonitrile (3j)

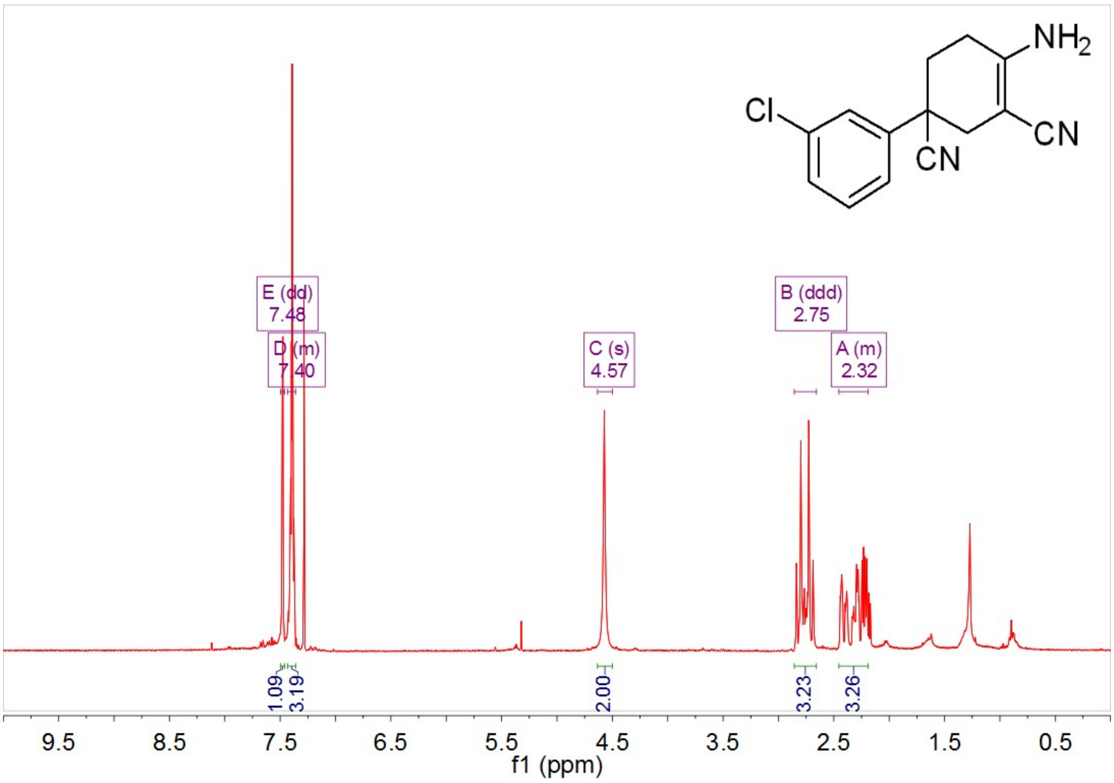

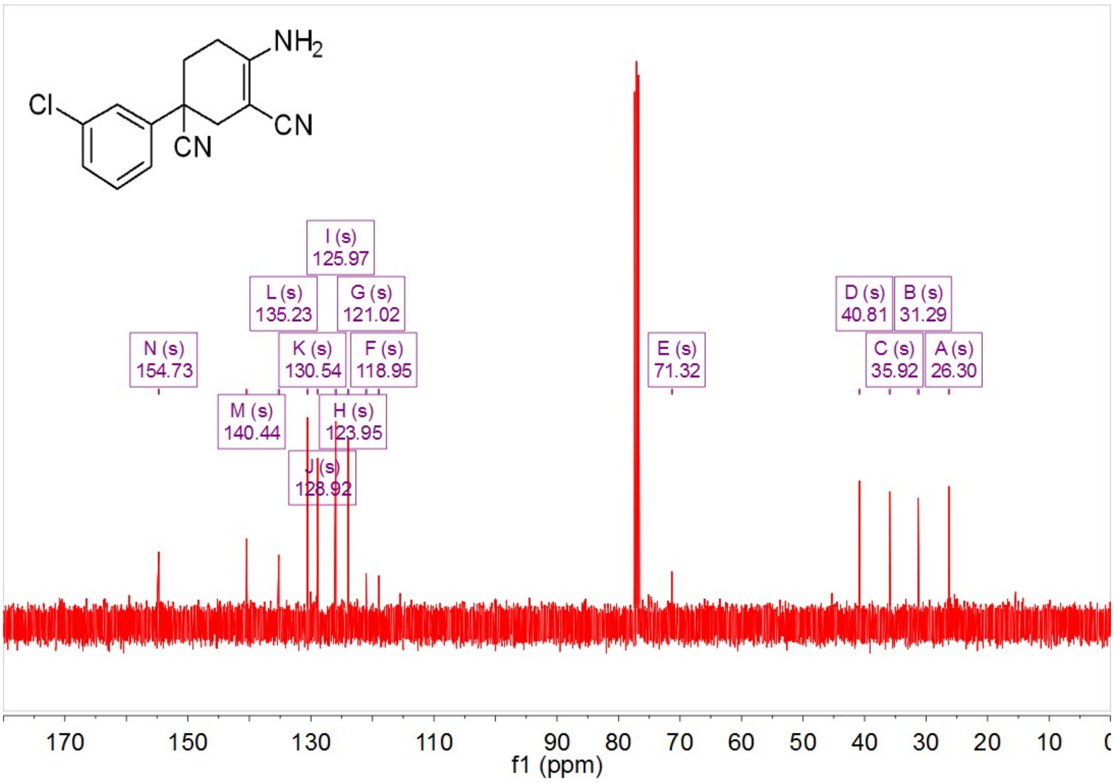

:

^{1}H NMR (400M, $CDCl_3$) and ^{13}C (101M, $CDCl_3$) of 4-amino-3'-fluoro-5,6-di

hydro-[1,1'-biphenyl]-1,3(2H)-dicarbonitrile (3k):

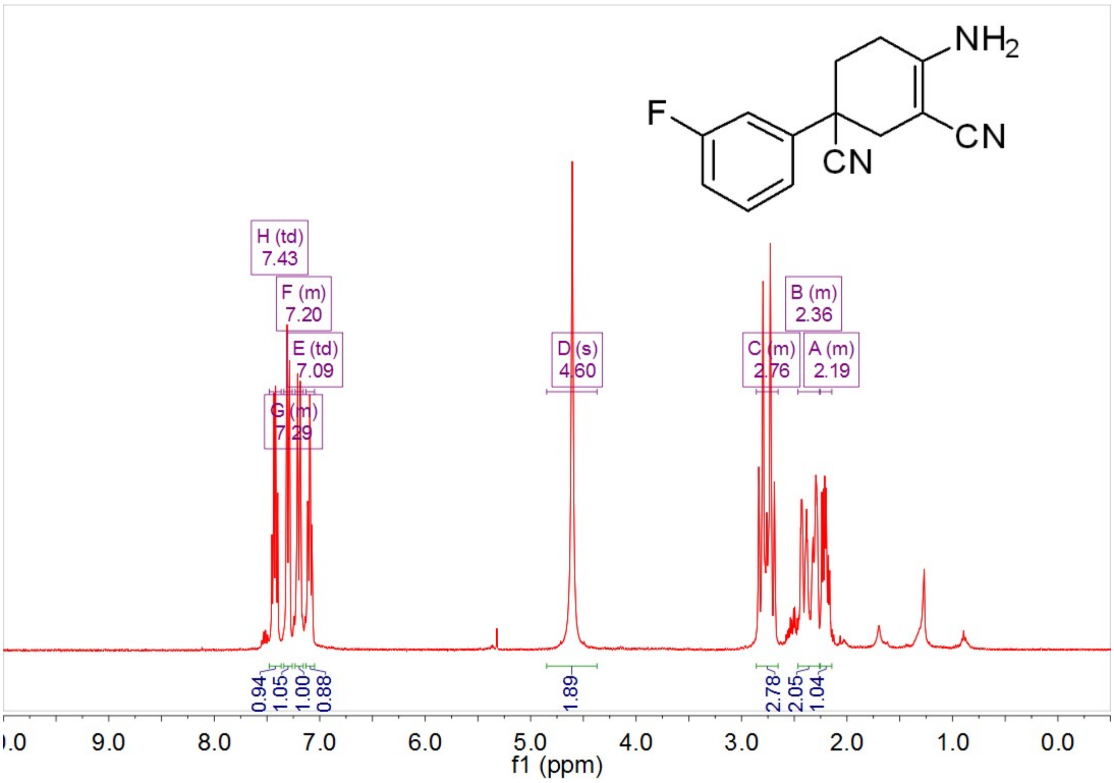

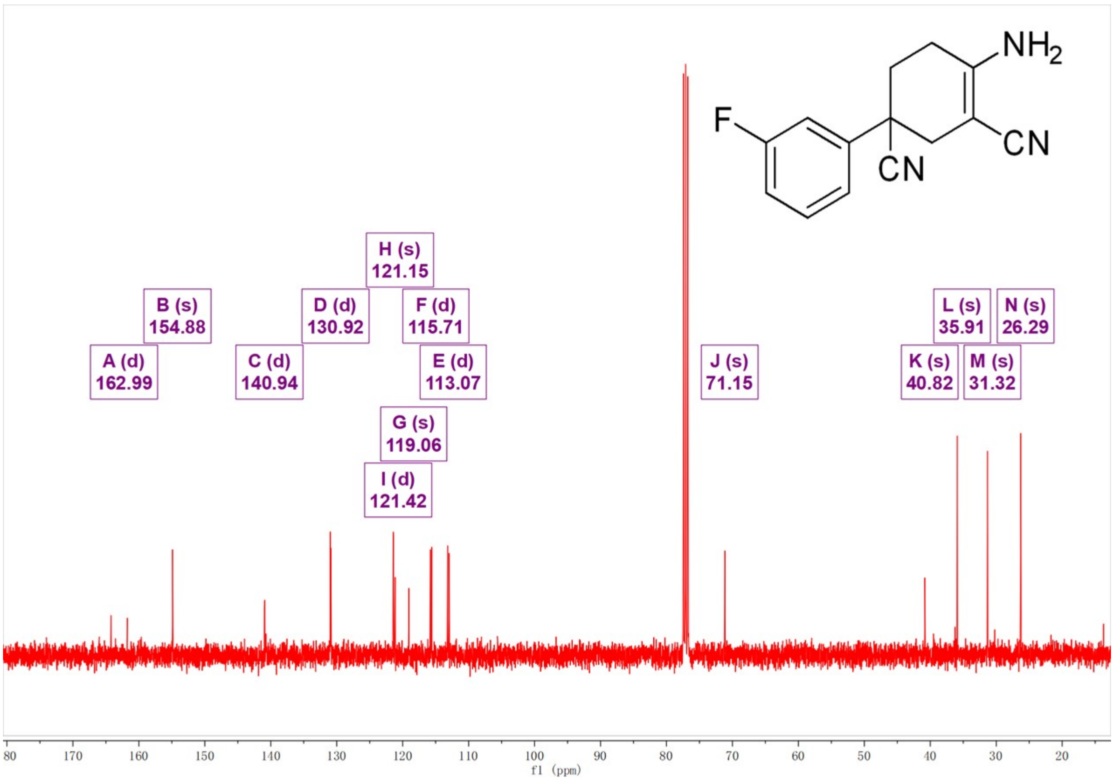

^{1}H NMR (400M, $CDCl_3$) and ^{13}C (101M, $CDCl_3$) of 4-amino-2'-methyl-5,6-d

ihydro-[1,1'-biphenyl]-1,3(2H)-dicarbonitrile (3l):

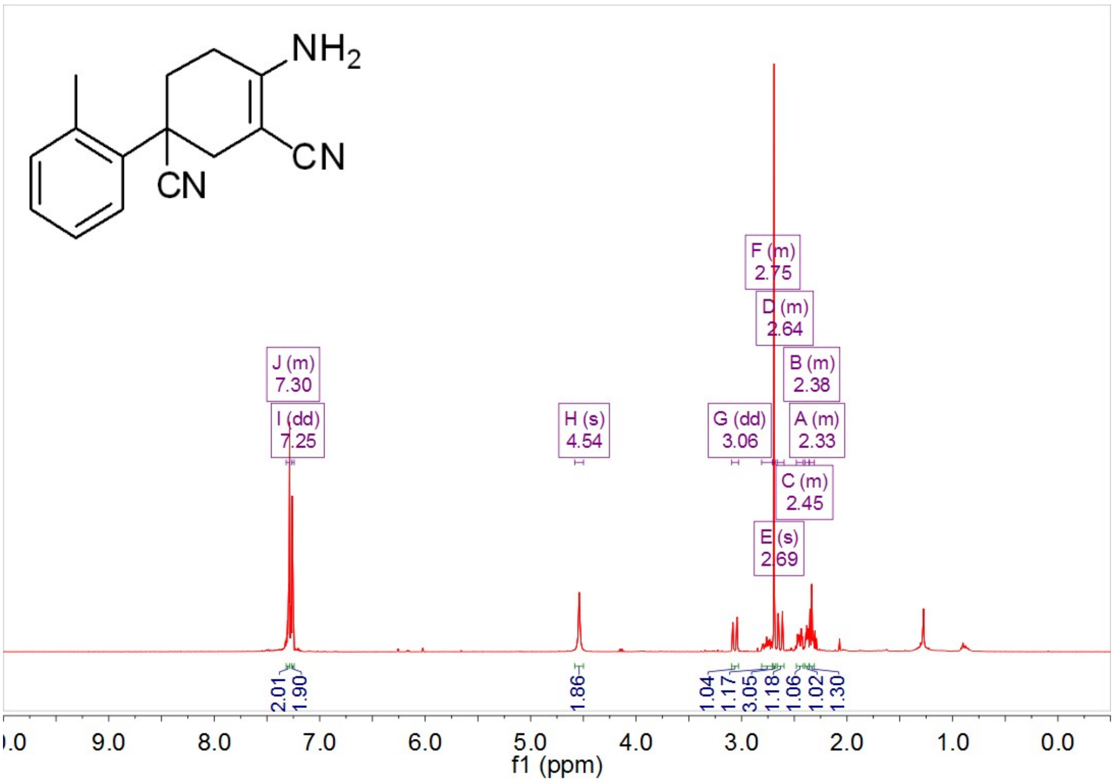

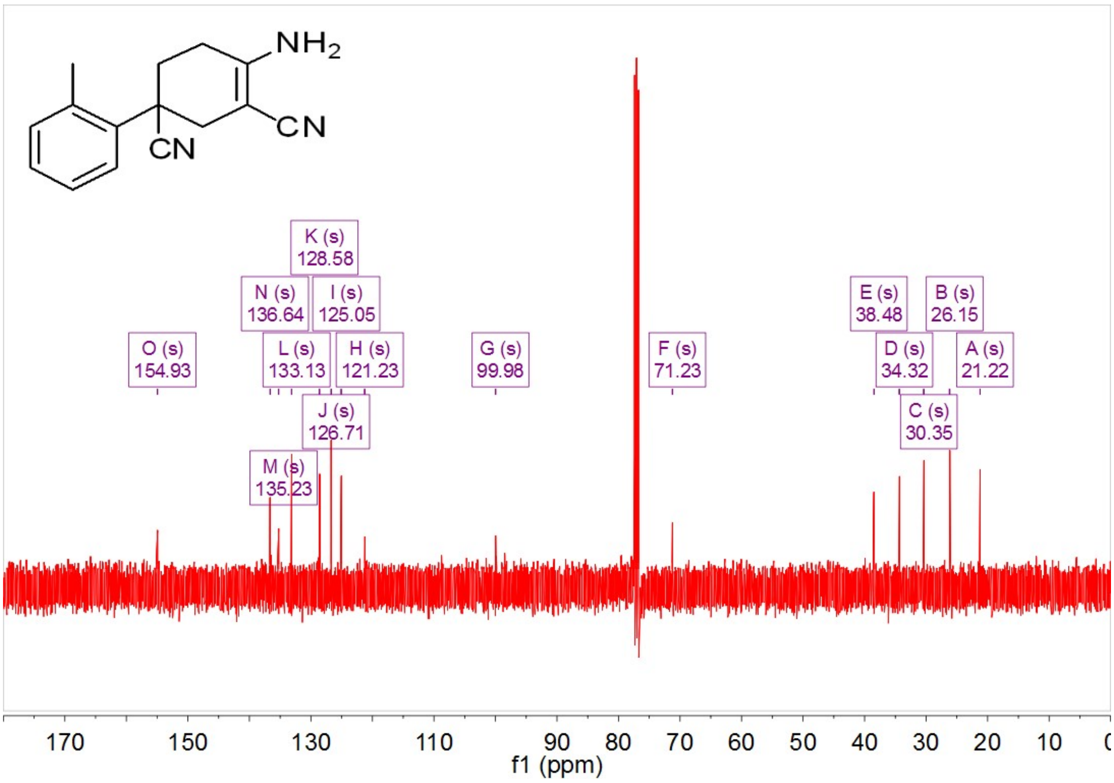

^{1}H NMR (400M, $CDCl_3$) and ^{13}C (101M, $CDCl_3$) of 4-amino-2'-chloro-5,6-di

hydro-[1,1'-biphenyl]-1,3(2H)-dicarbonitrile (3m):

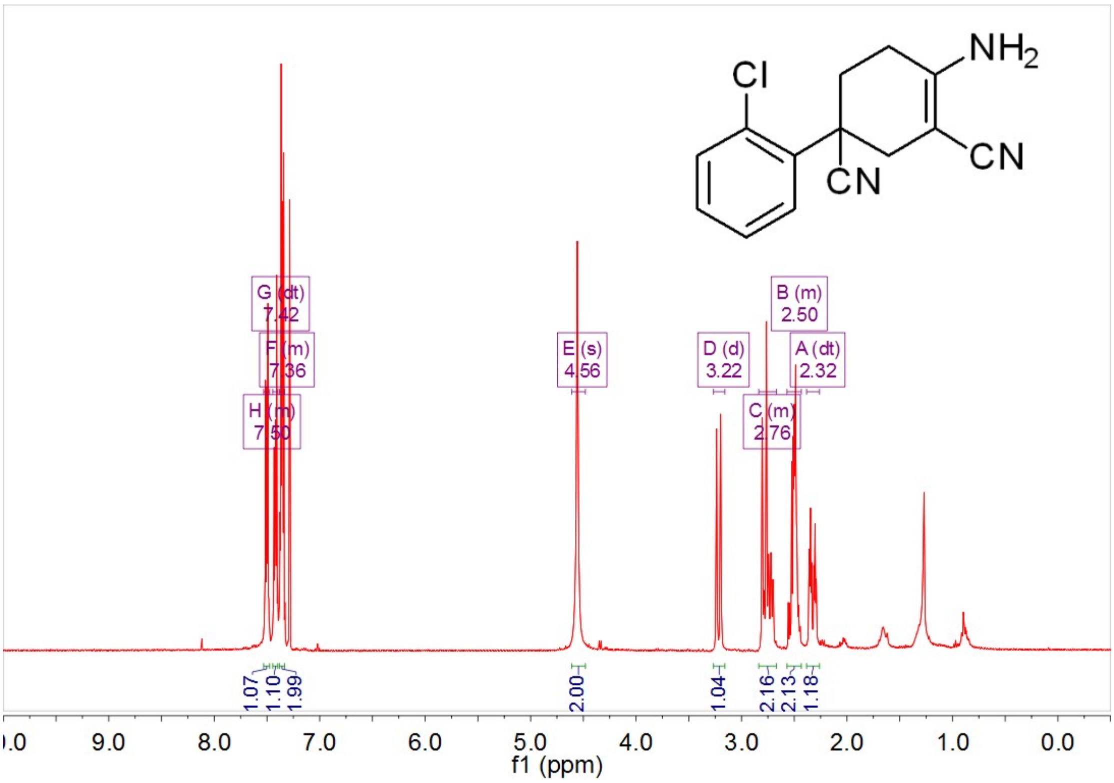

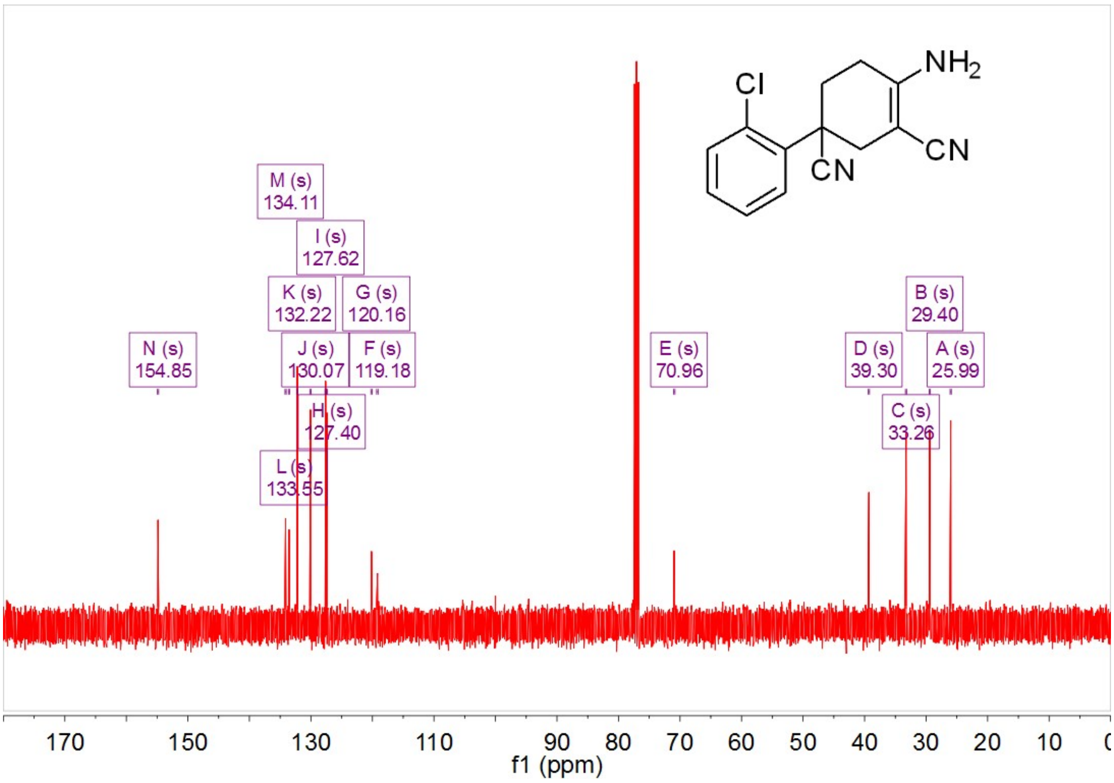

^{1}H NMR (400M, $CDCl_3$) and ^{13}C (101M, $CDCl_3$) of 4-amino-2'-fluoro-5,6-di

hydro-[1,1'-biphenyl]-1,3(2H)-dicarbonitrile (3n):

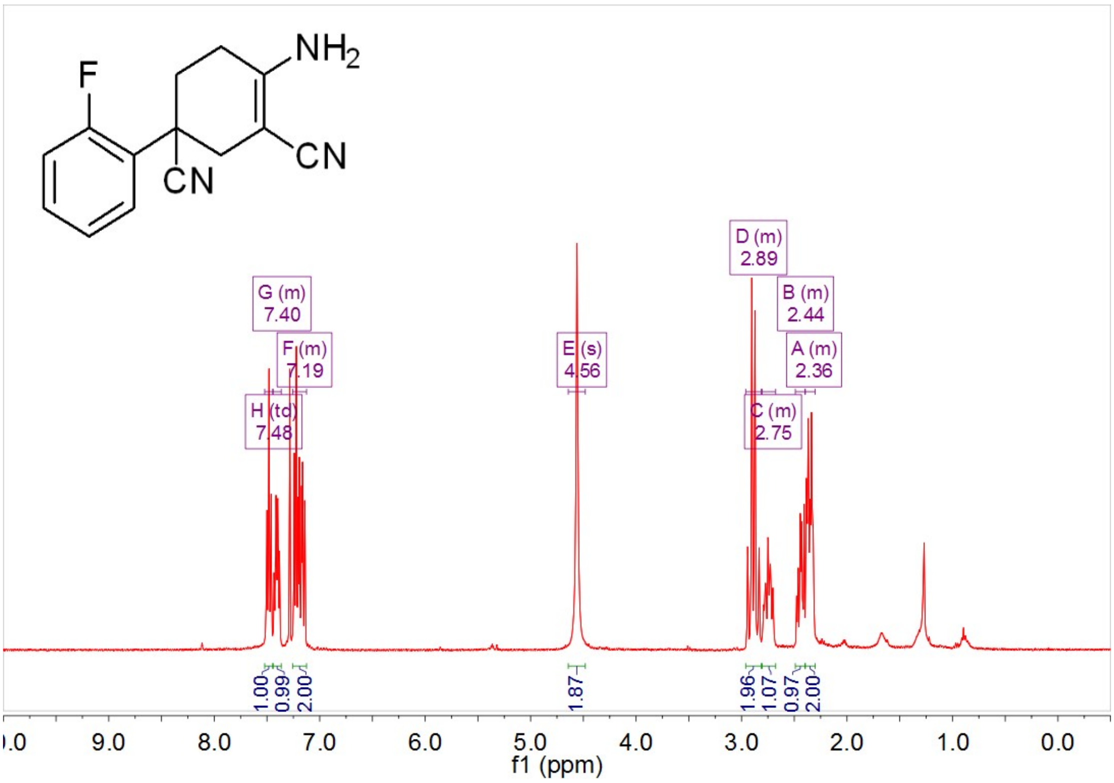

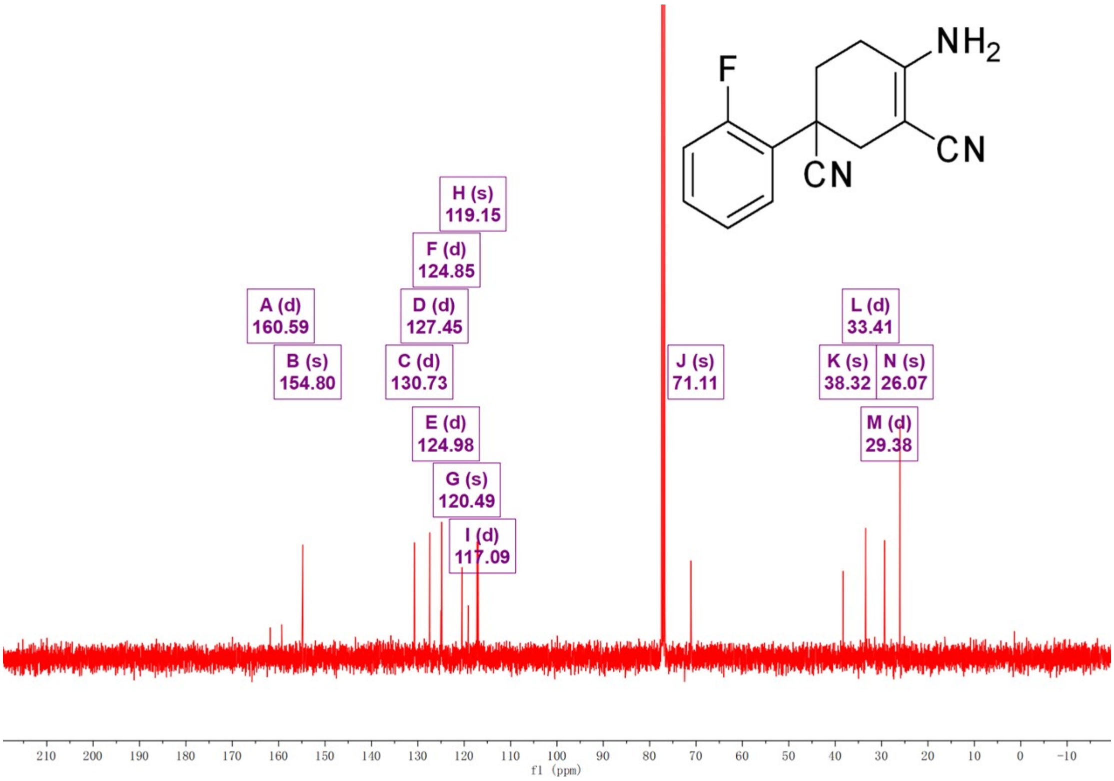

^{1}H NMR (400M, $CDCl_3$) and ^{13}C (101M, $CDCl_3$) of 4-amino-3',4'-dimethoxy

-5,6-dihydro-[1,1'-biphenyl]-1,3(2H)-dicarbonitrile (3o):

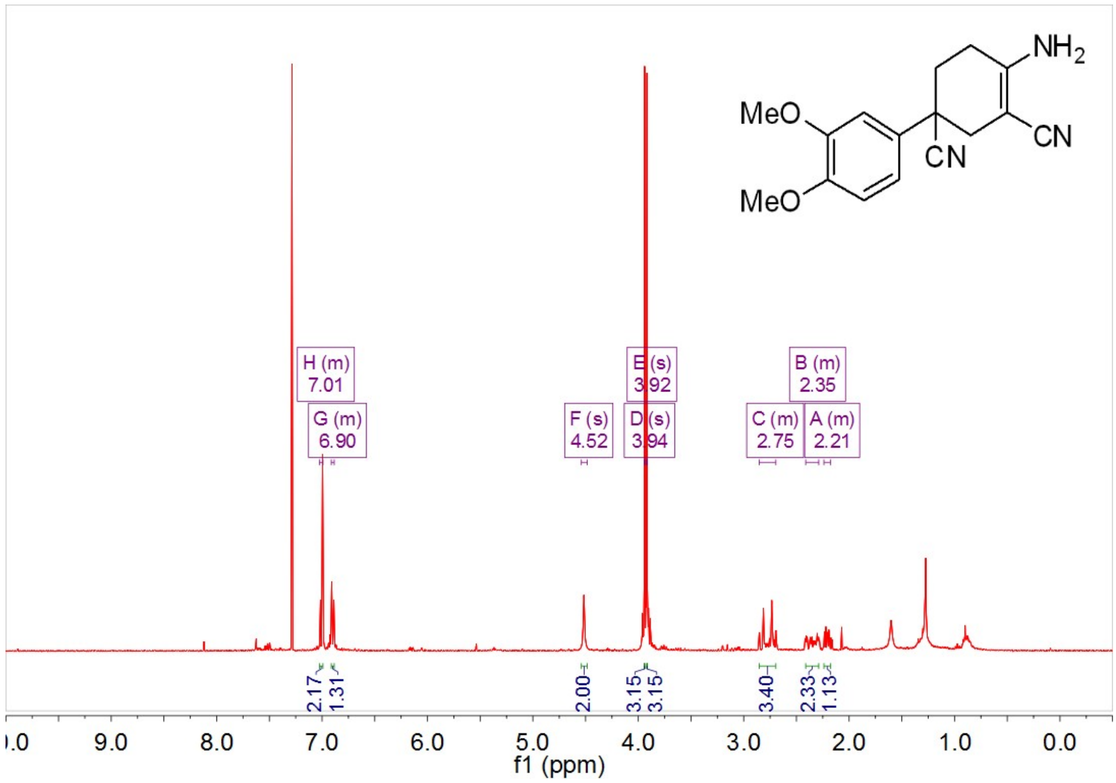

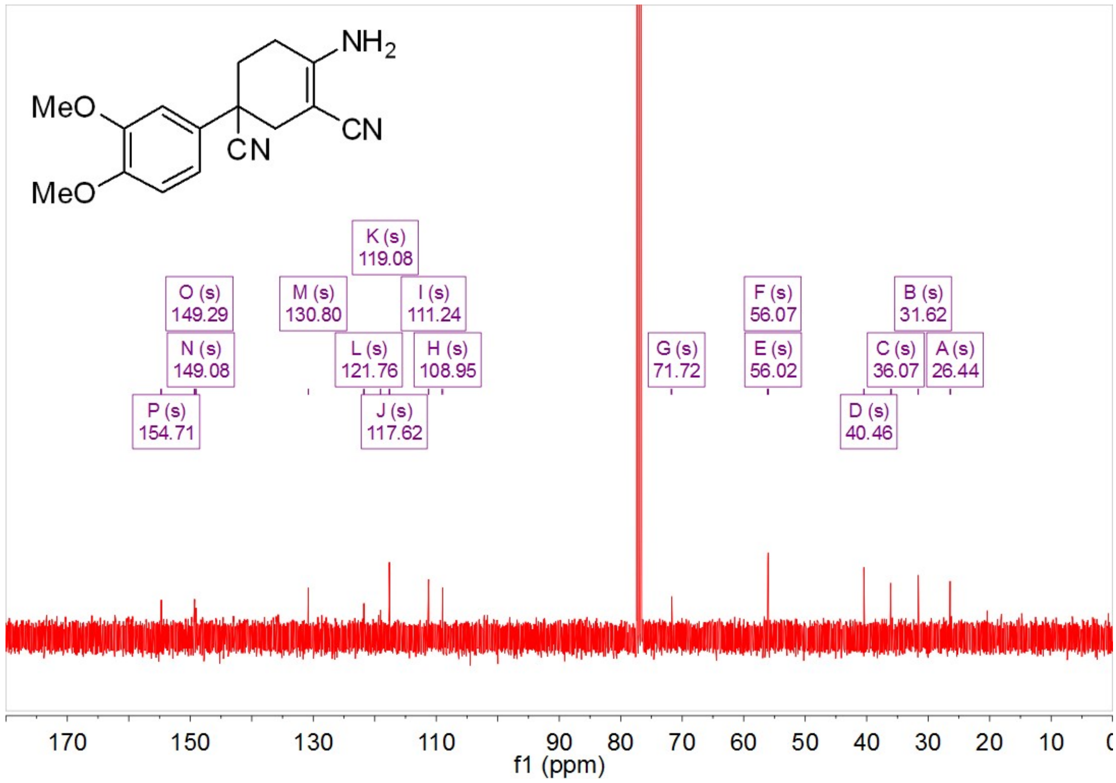

^{1}H NMR (400M, $CDCl_3$) and ^{13}C (101M, $CDCl_3$) of 4-amino-1-(benzo[d][1,3]d

ioxol-5-yl)cyclohex-3-ene-1,3-dicarbonitrile (3p):

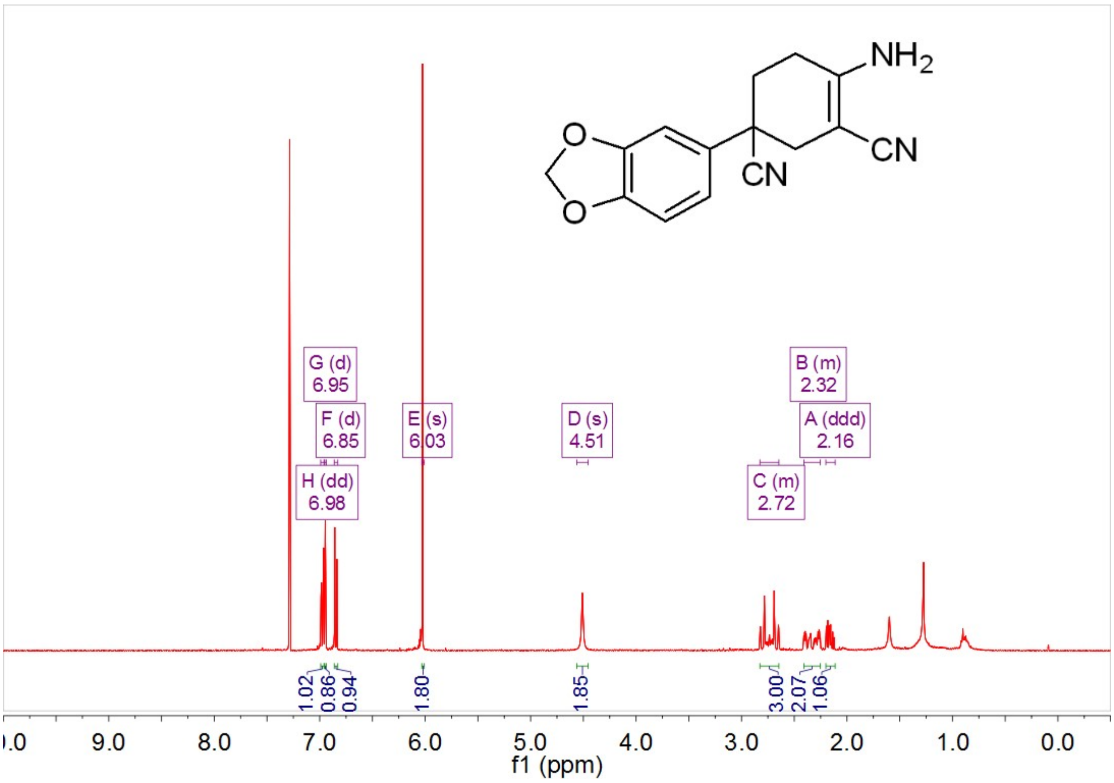

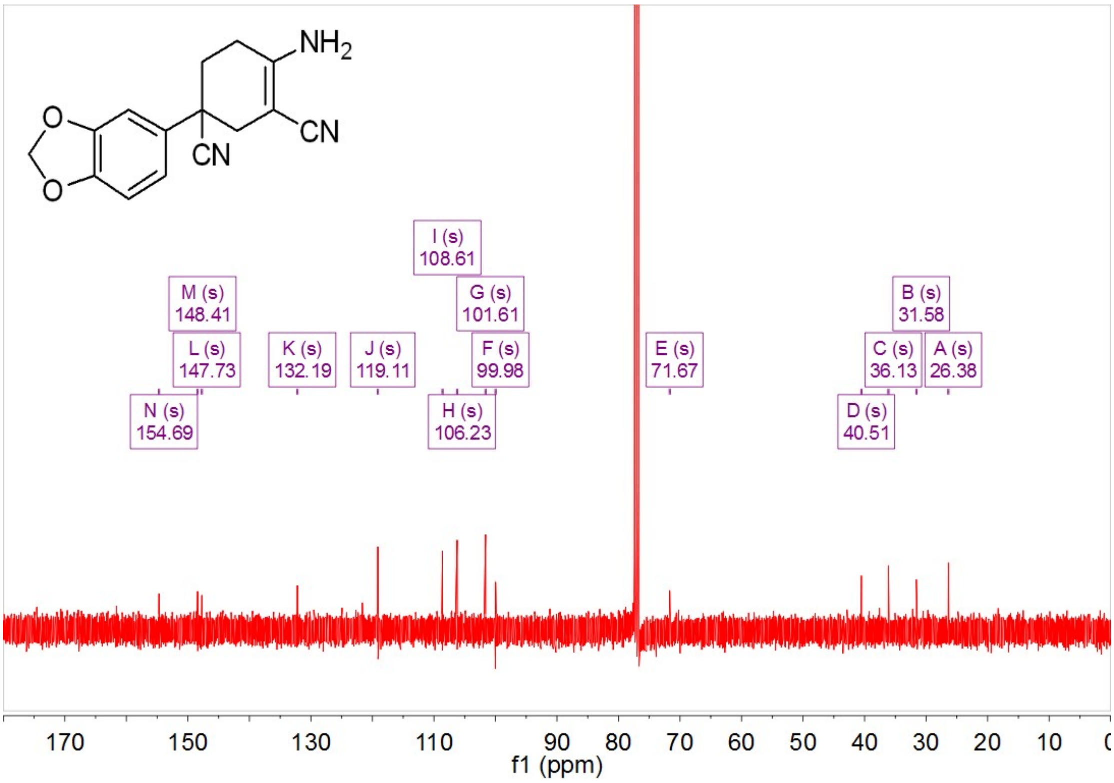

^{1}H NMR (400M, $CDCl_3$) and ^{13}C (101M, $CDCl_3$) of 4-amino-1-(pyridin-2-yl)

cyclohex-3-ene-1,3-dicarbonitrile (3q):

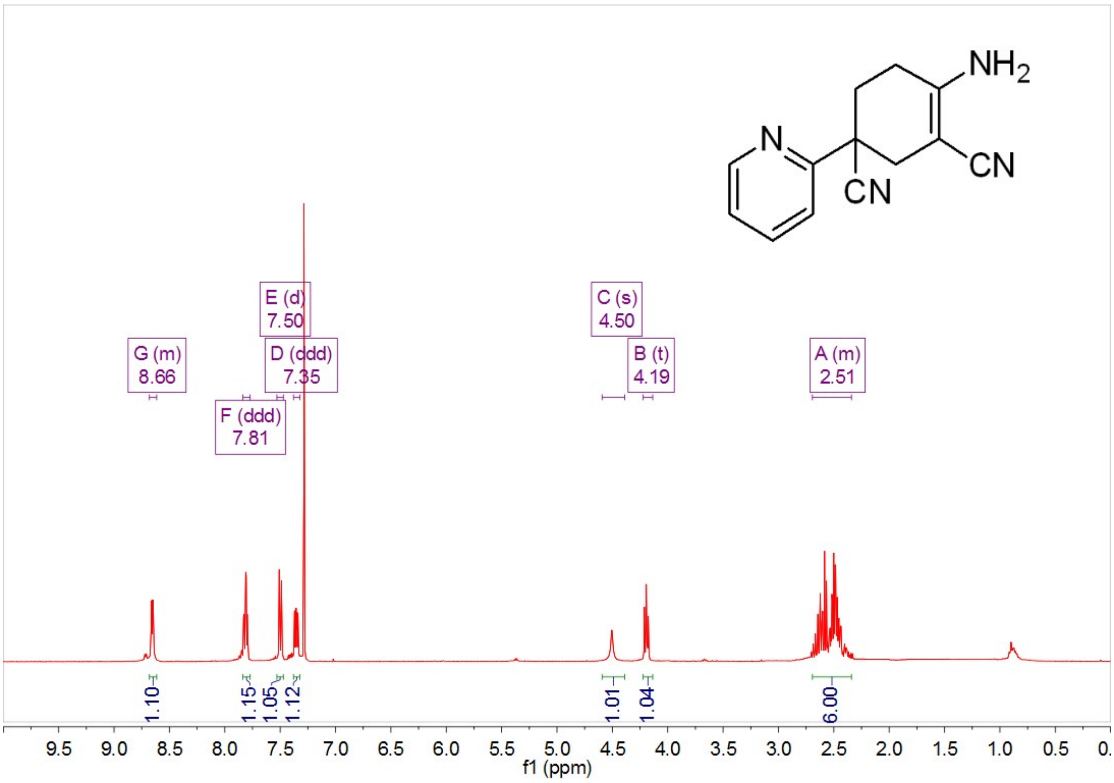

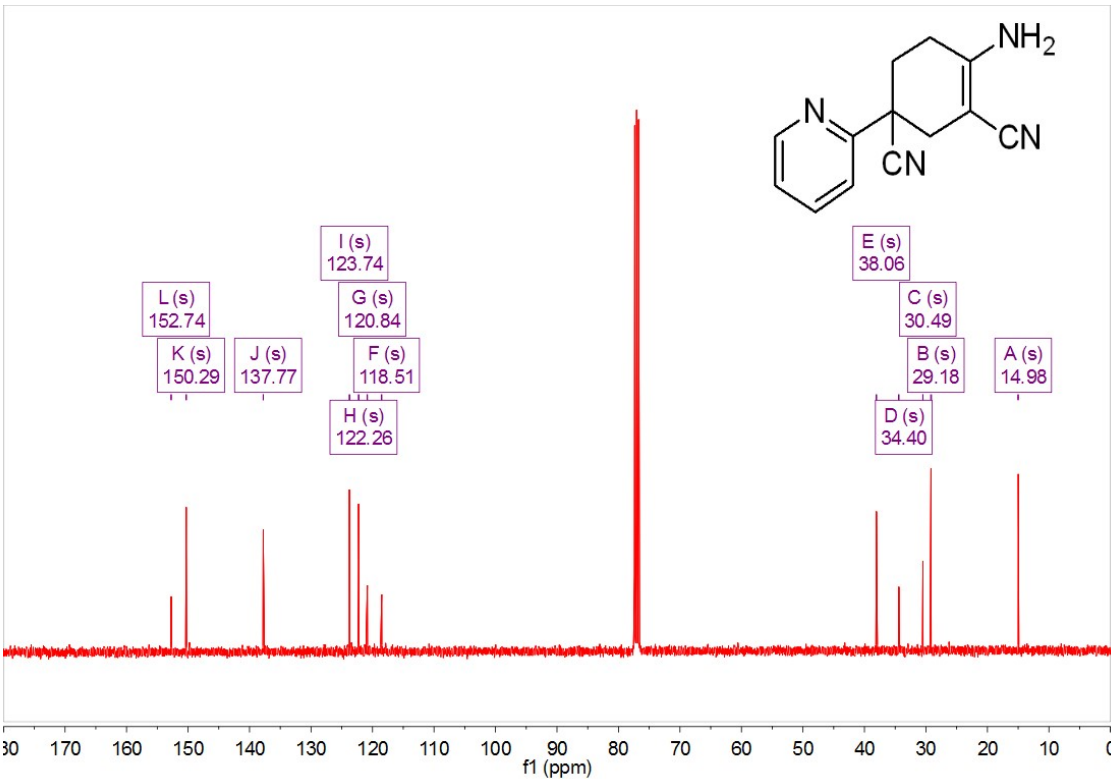

^{1}H NMR (400M, $CDCl_3$) and ^{13}C (101M, $CDCl_3$) of 4-amino-1-(pyridin-3-yl)

cyclohex-3-ene-1,3-dicarbonitrile (3r):

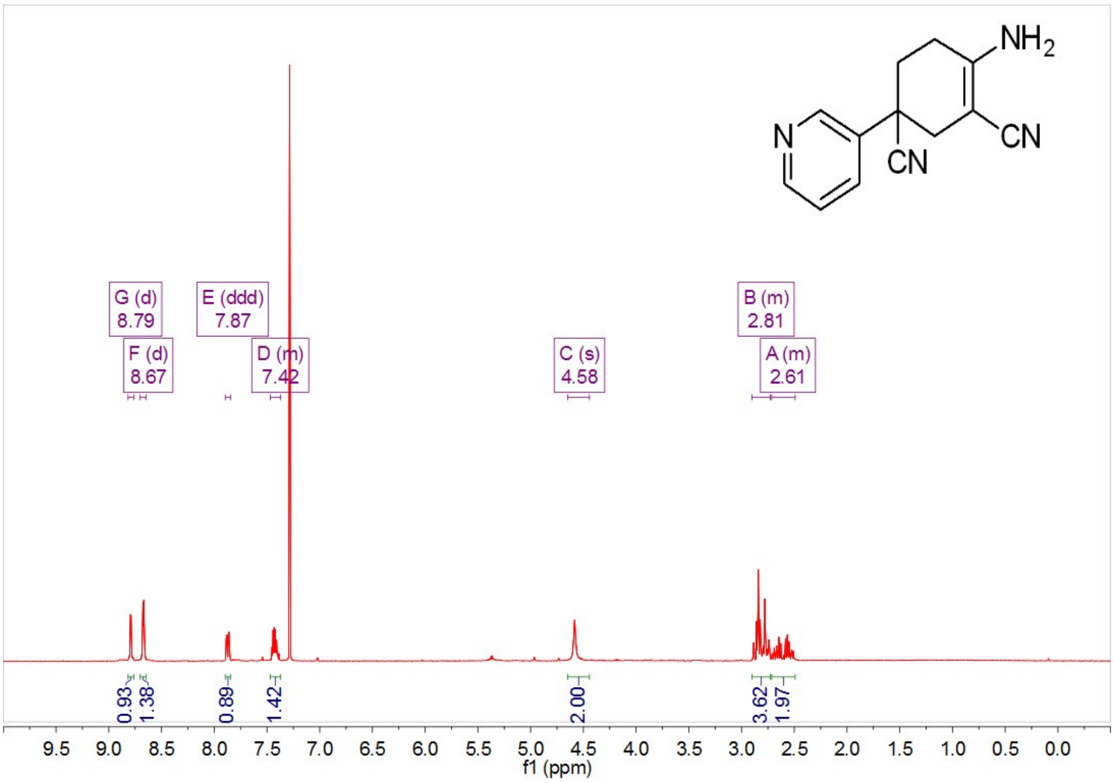

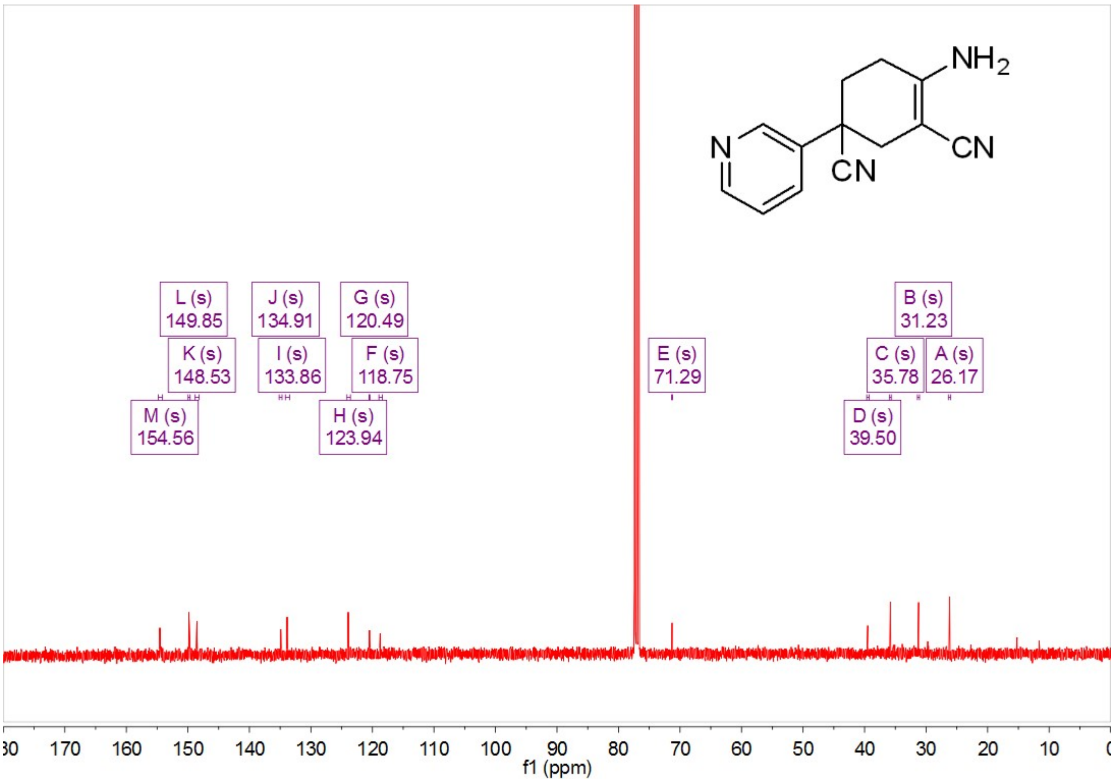

^{1}H NMR (400M, $CDCl_3$) and ^{13}C (101M, $CDCl_3$) of 4-amino-1-(naphthalen-

2-yl)cyclohex-3-ene-1,3-dicarbonitrile (3s):

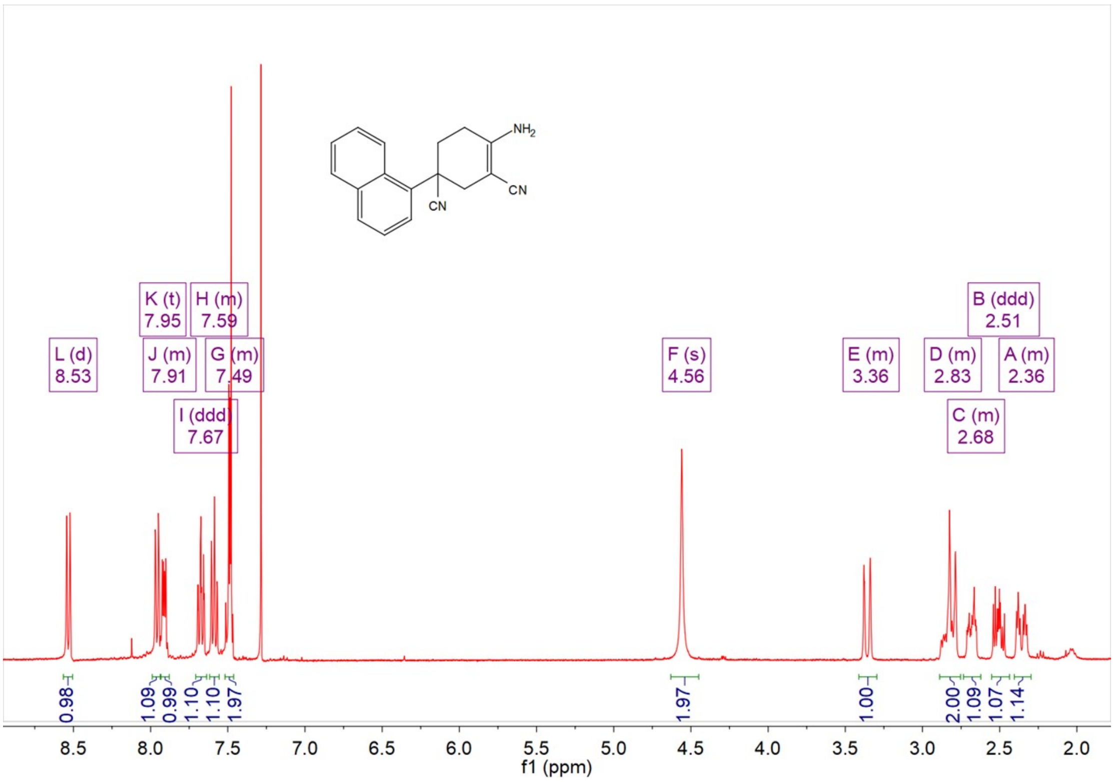

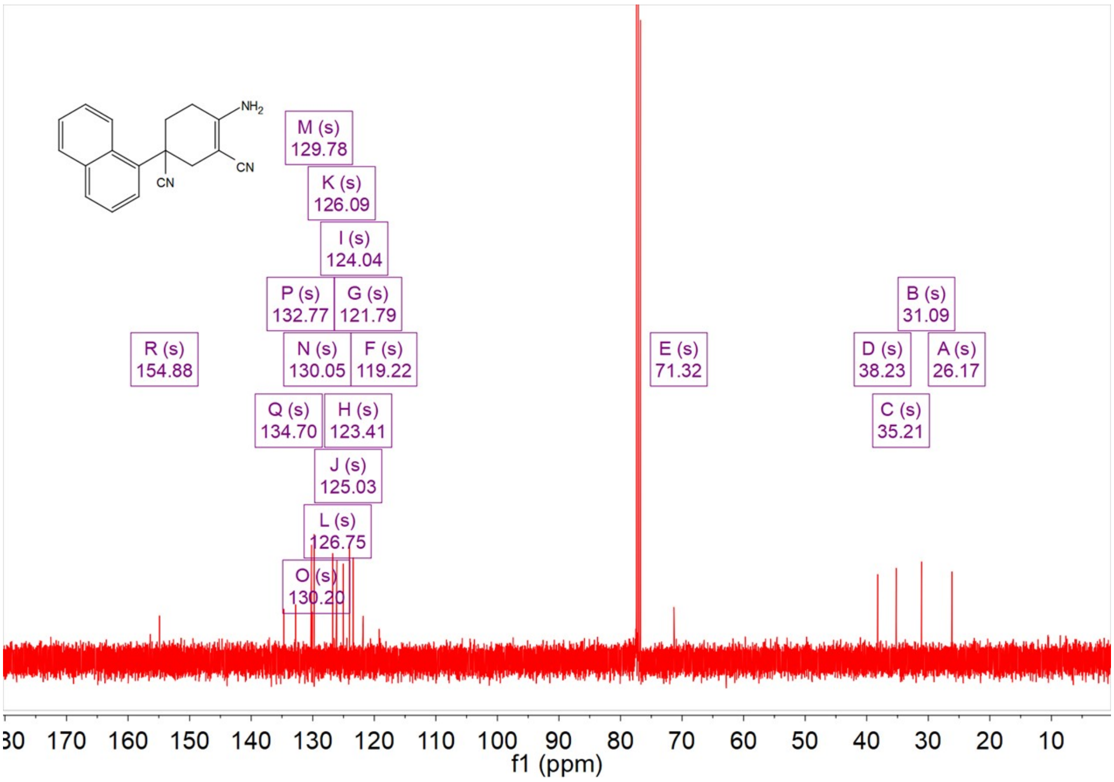

^{1}H NMR (400M, $CDCl_3$) and ^{13}C (101M, $CDCl_3$) of 4-amino-4'-ethynyl-5,6-

dihydro-[1,1'-biphenyl]-1,3(2H)-dicarbonitrile (3t):

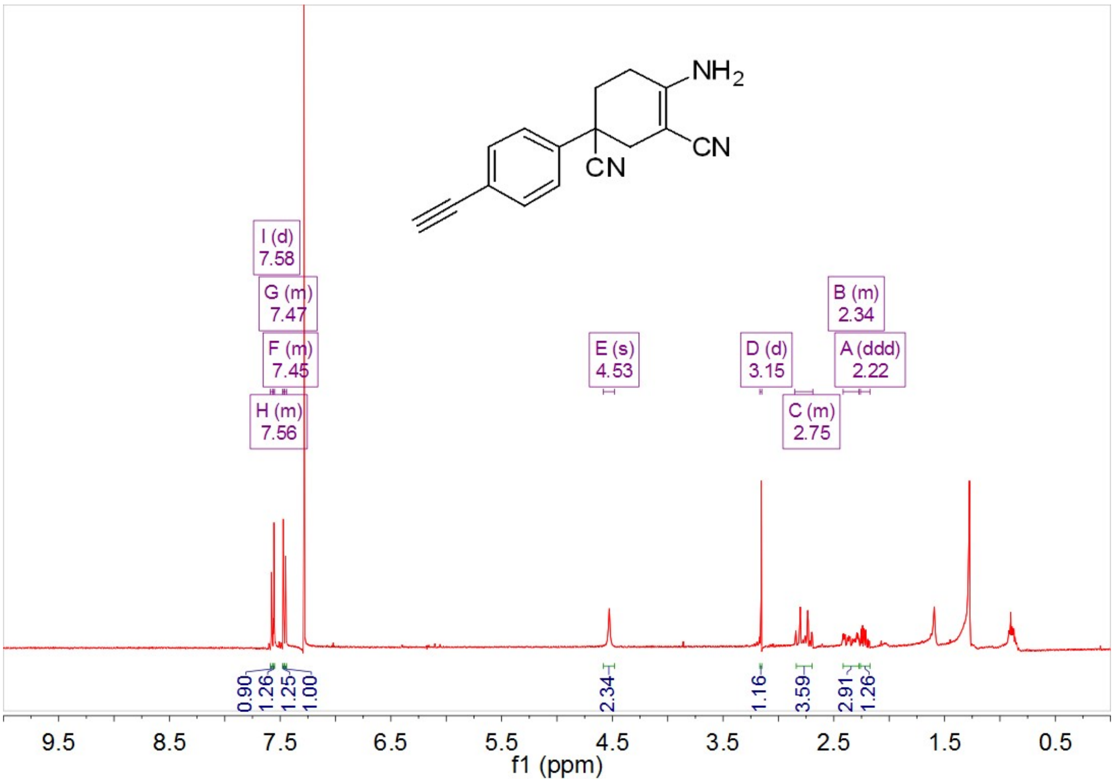

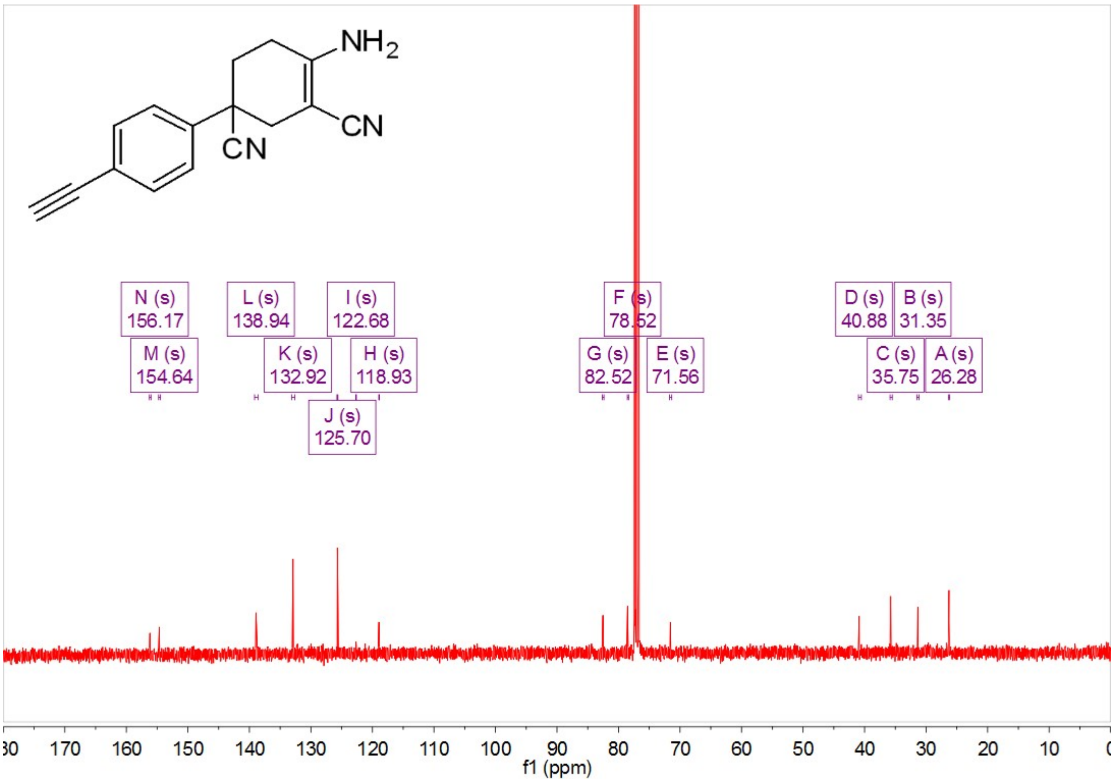

^{1}H NMR (400M, $CDCl_3$) and ^{13}C (101M, $CDCl_3$) of 3-(2-fluorophenyl)penta

ne-1,3,5-tricarbonitrile (3n'):

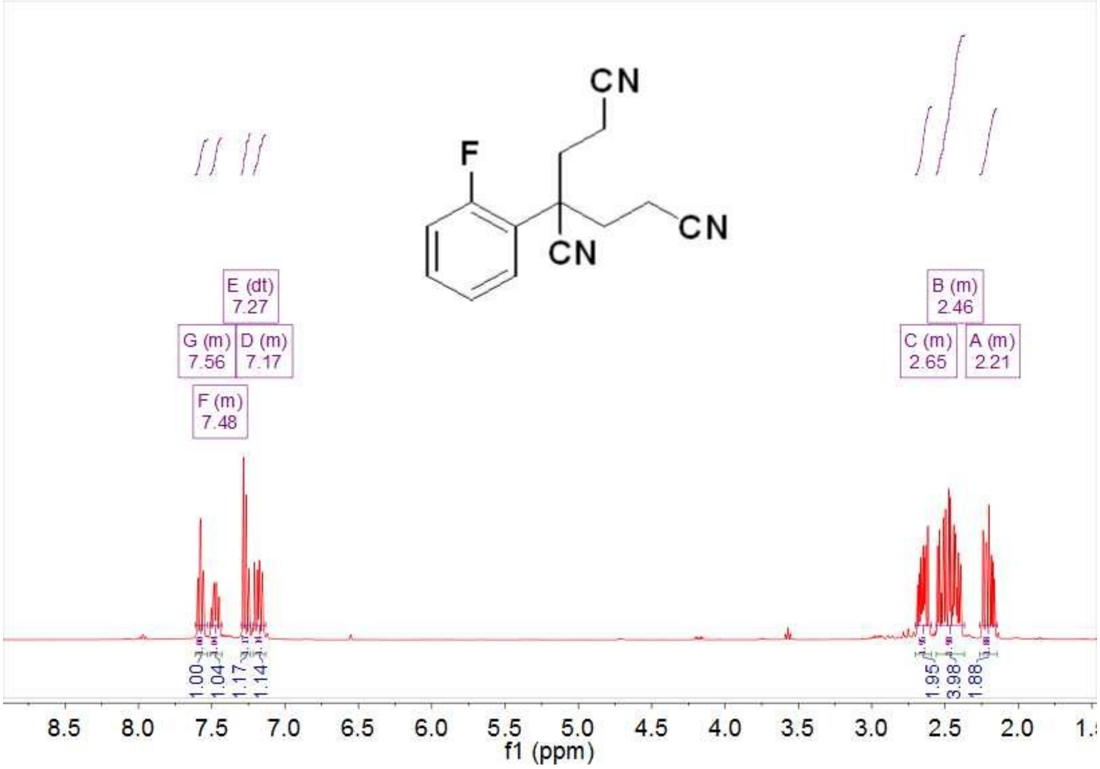

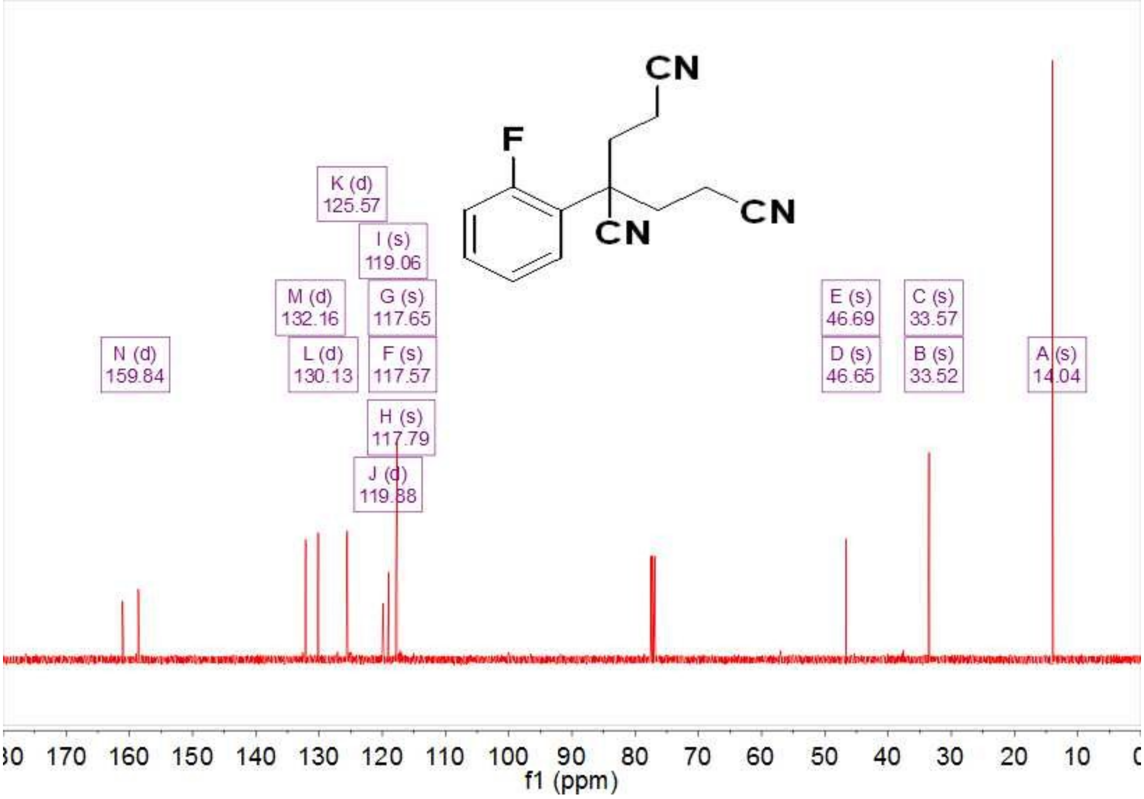

^{1}H NMR (400M, $CDCl_3$) and ^{13}C (101M, $CDCl_3$) of 4-amino-2,6-diphenyl-5,

6,7,8-tetrahydroquinazoline-6-carbonitrile (5a):

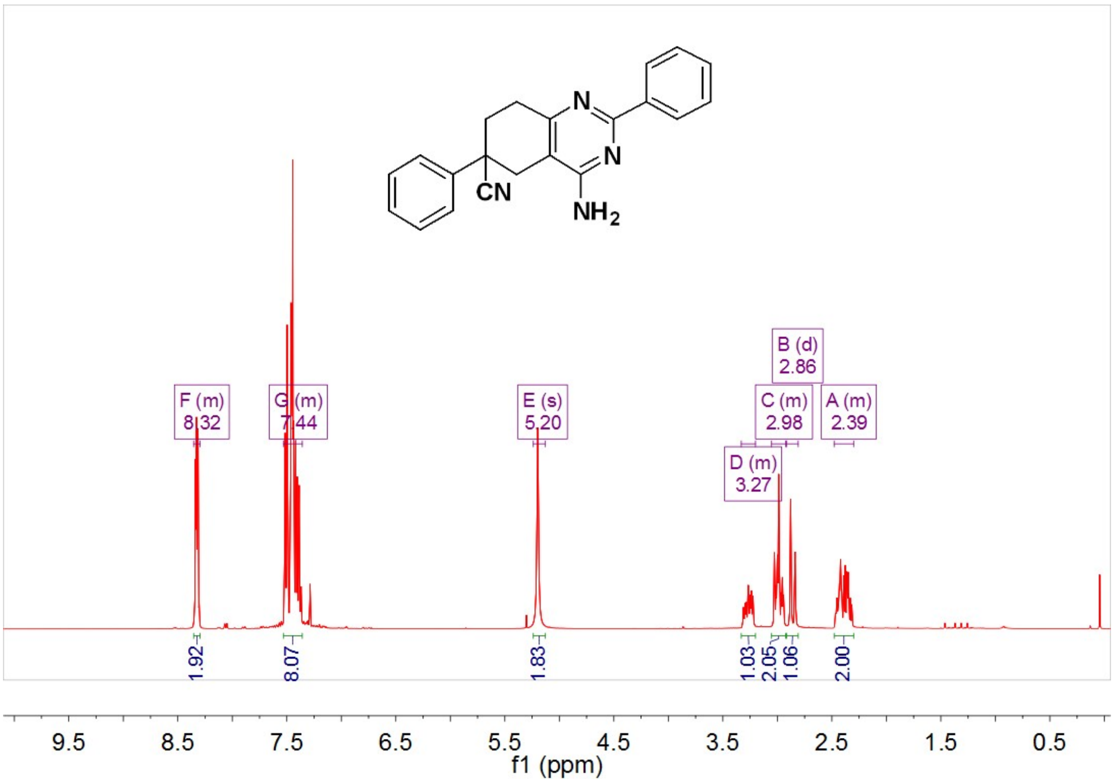

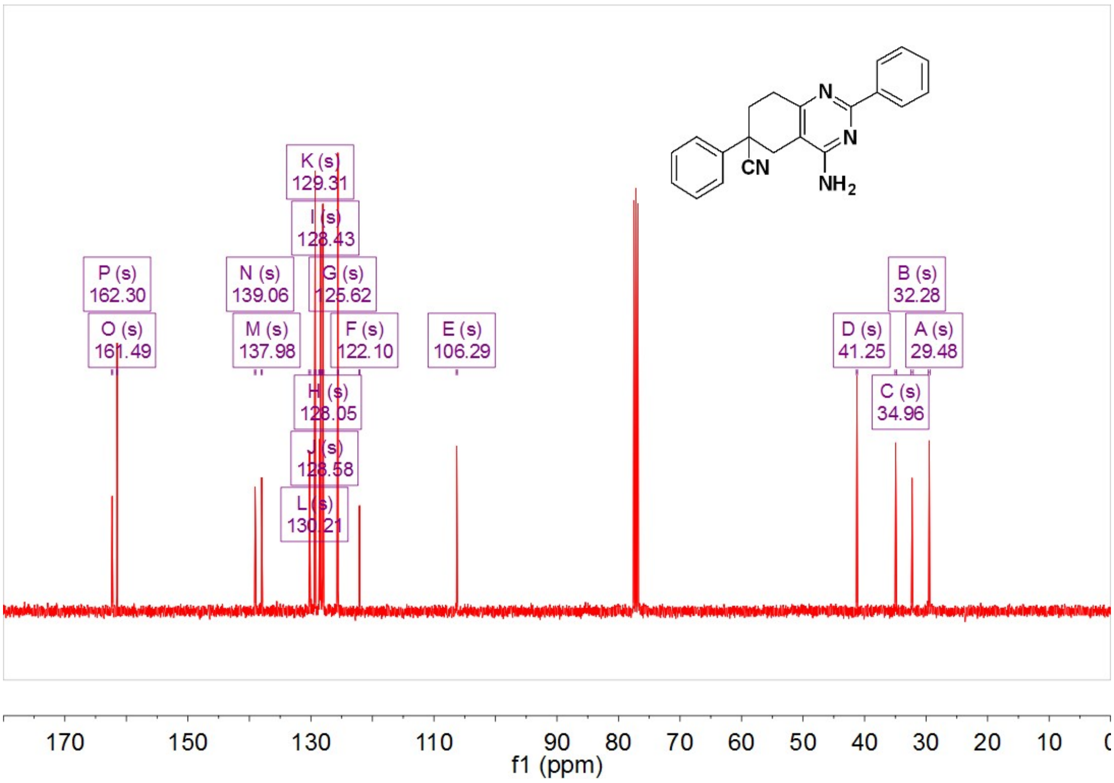

^{1}H NMR (400M, CDCl$_3$) and ^{13}C (101M, CDCl$_3$) of 4-amino-6-phenyl-2-(p-t

olyl)-5,6,7,8-tetrahydroquinazoline-6-carbonitrile (5b):

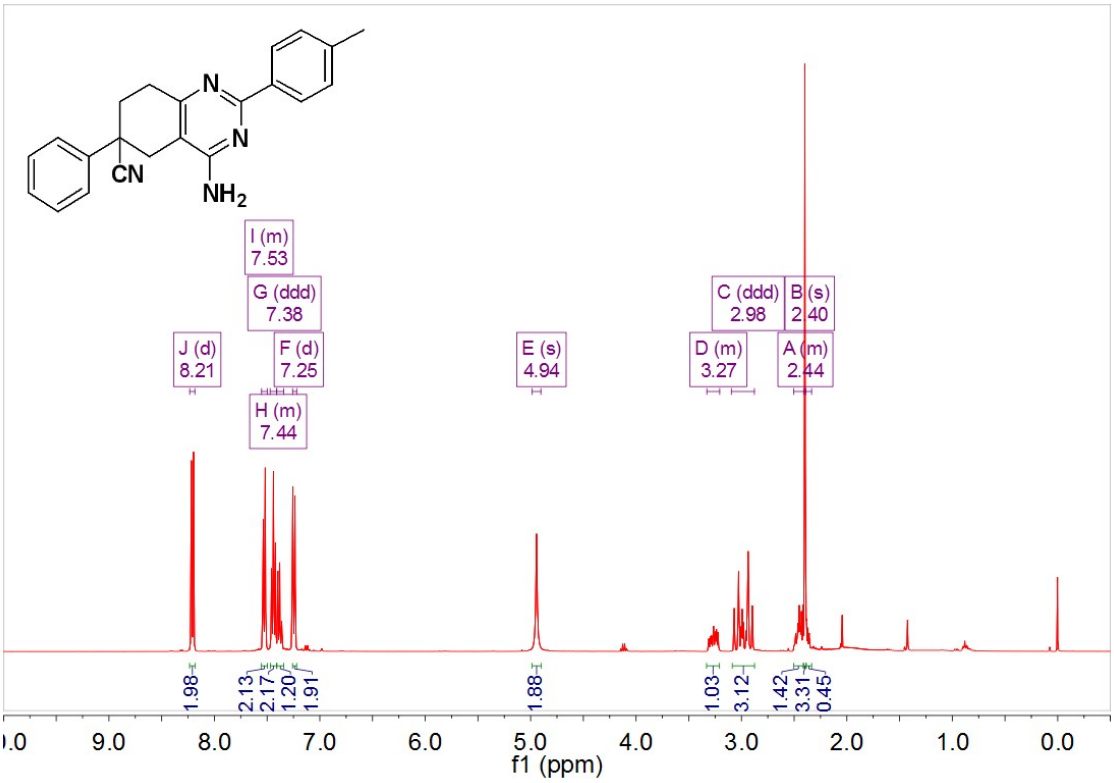

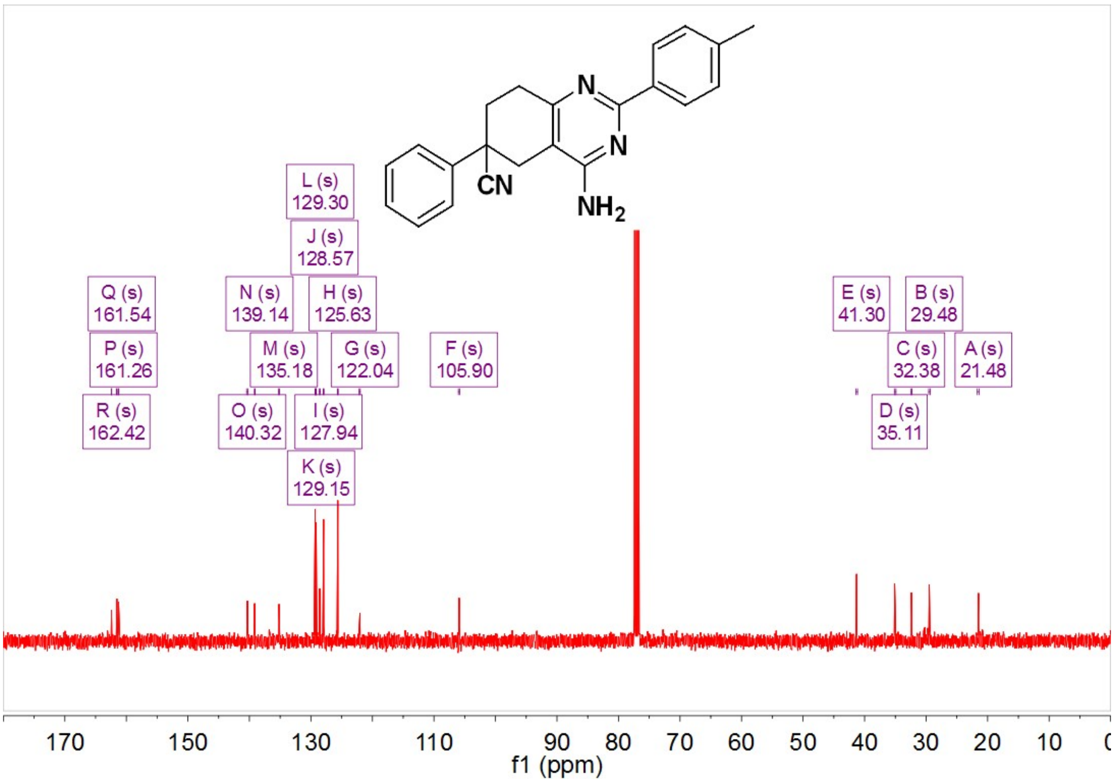

^{1}H NMR (400M, $CDCl_3$) and ^{13}C (101M, $CDCl_3$) of 4-amino-6-phenyl-2-(4-(

trifluoromethyl)phenyl)-5,6,7,8-tetrahydroquinazoline-6-carbonitrile (5c):

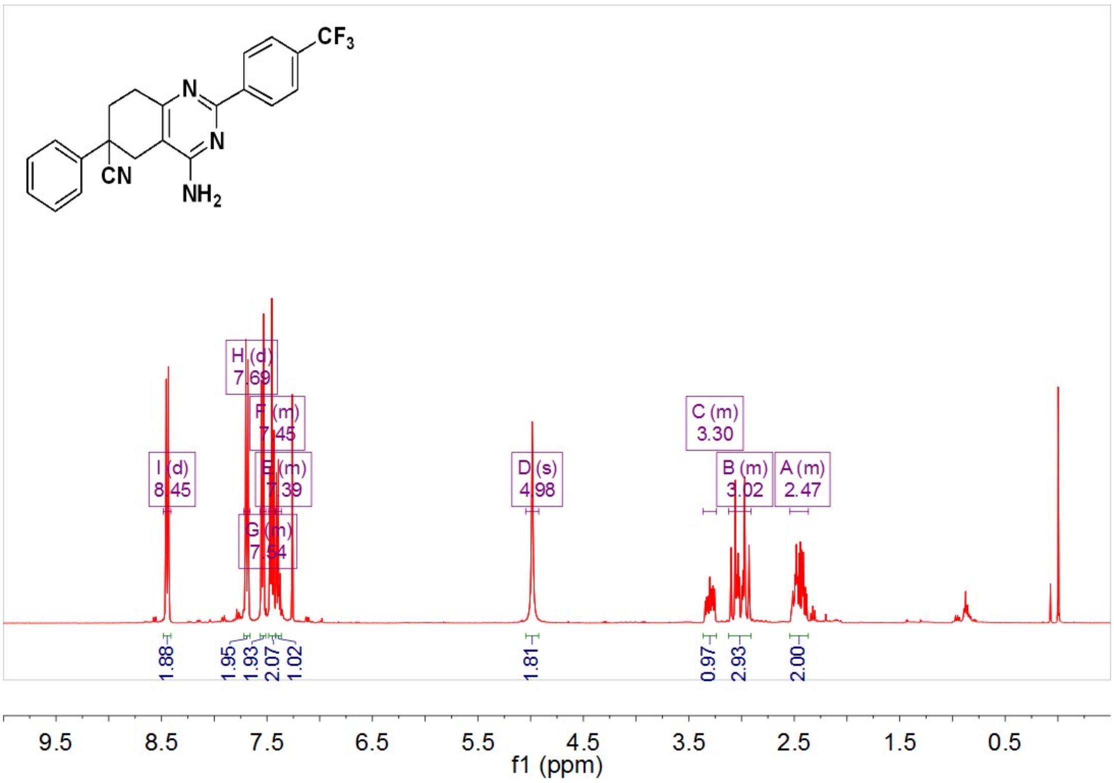

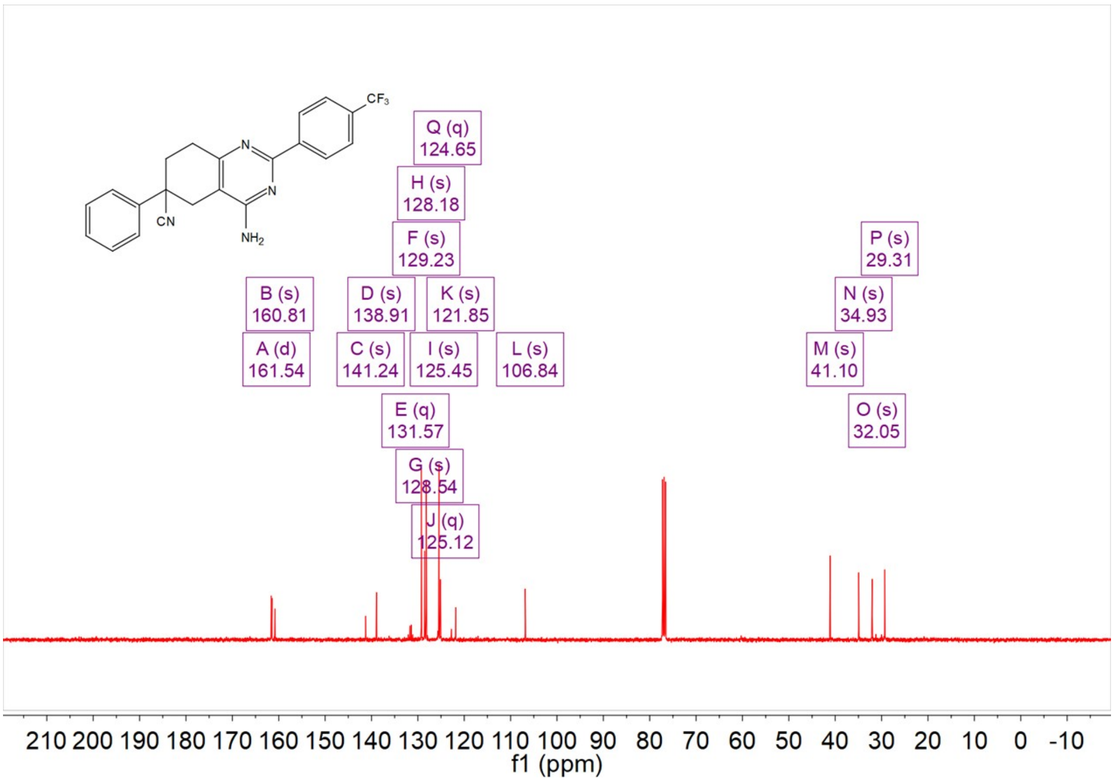

^{1}H NMR (400M, $CDCl_3$) and ^{13}C (101M, $CDCl_3$) of 4-amino-2-(naphthalen-

2-yl)-6-phenyl-5,6,7,8-tetrahydroquinazoline-6-carbonitrile (5d)::

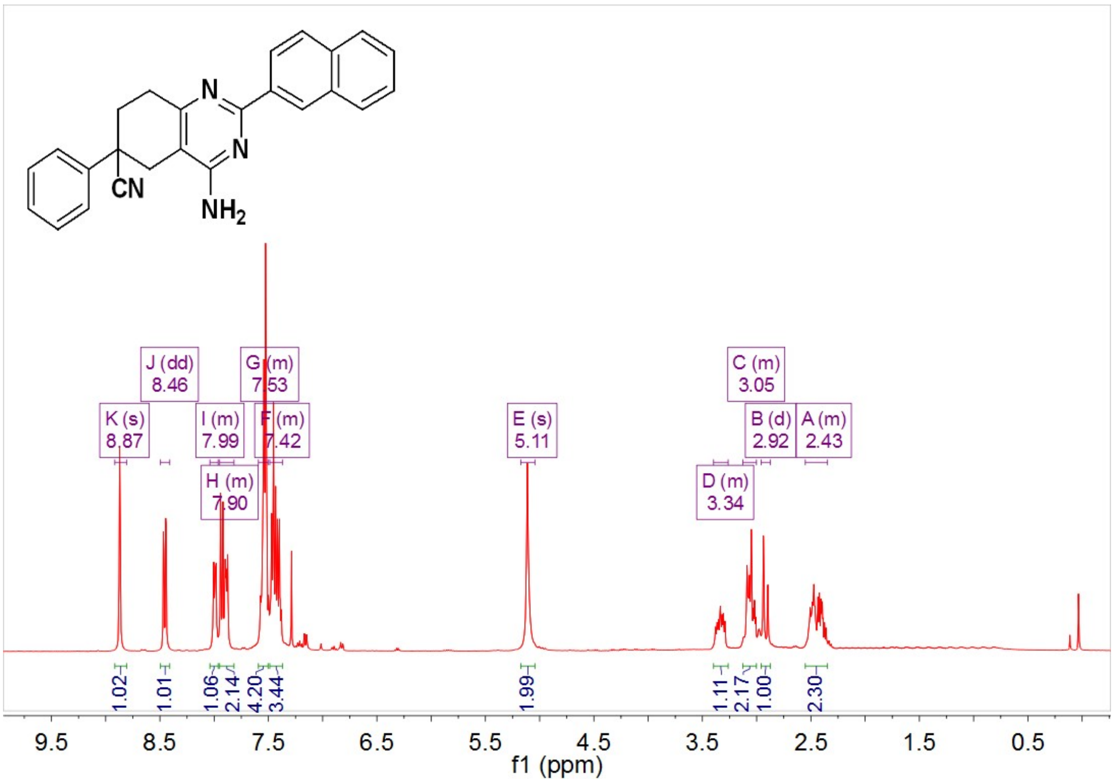

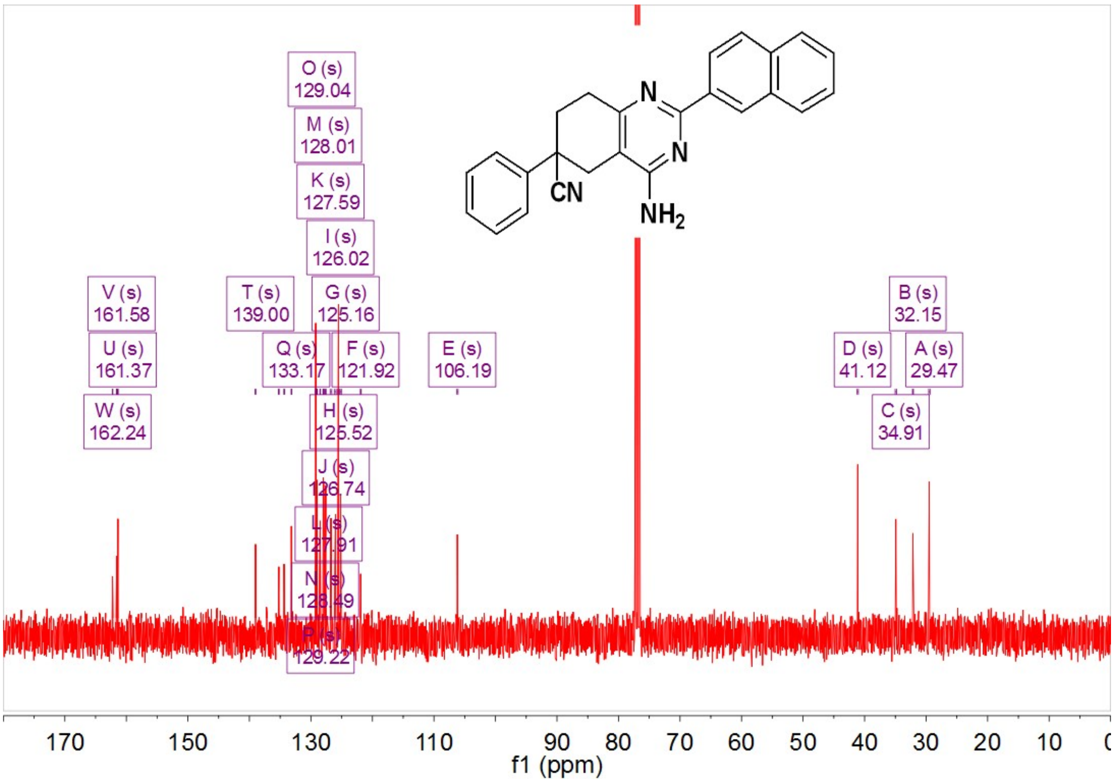

7. References

1. CrysAlisPro, Oxford Diffraction (Poland),2010.

2. a) G. M. Sheldrick, SHELXS-97, Program for the Solution of Crystal Structure.

University of Göttingen, Germany 1997. b) G. M. Sheldrick, *Acta Crystallogr.*,2015, **C71**, 3.
